# The proportion of endometrial tumours associated with Lynch syndrome (PETALS): A prospective cross-sectional study

**DOI:** 10.1371/journal.pmed.1003263

**Published:** 2020-09-17

**Authors:** Neil A. J. Ryan, Raymond McMahon, Simon Tobi, Tristan Snowsill, Shona Esquibel, Andrew J. Wallace, Sancha Bunstone, Naomi Bowers, Ioana E. Mosneag, Sarah J. Kitson, Helena O’Flynn, Neal C. Ramchander, Vanitha N. Sivalingam, Ian M. Frayling, James Bolton, Rhona J. McVey, D. Gareth Evans, Emma J. Crosbie

**Affiliations:** 1 Division of Cancer Sciences, Faculty of Biology, Medicine and Health, University of Manchester, St Mary's Hospital, Manchester, United Kingdom; 2 Division of Evolution and Genomic Medicine, University of Manchester, St Mary's Hospital, Manchester, United Kingdom; 3 Department of Pathology, Manchester University NHS Foundation Trust, Manchester Academic Health Science Centre, Manchester, United Kingdom; 4 Manchester Centre for Genomic Medicine, North-West Genomics Laboratory Hub, Manchester University NHS Foundation Trust, Manchester Academic Health Science Centre, Manchester, United Kingdom; 5 Health Economics Group, University of Exeter Medical School, University of Exeter, Exeter, Devon, United Kingdom; 6 Inherited Tumour Syndromes Research Group, Institute of Cancer & Genetics, Cardiff University, Cardiff, United Kingdom; 7 Department of Obstetrics and Gynaecology, St Mary’s Hospital, Manchester University NHS Foundation Trust, Manchester Academic Health Science Centre, Manchester, United Kingdom; Memorial University of Newfoundland Research, CANADA

## Abstract

**Background:**

Lynch syndrome (LS) predisposes to endometrial cancer (EC), colorectal cancer, and other cancers through inherited pathogenic variants affecting mismatch-repair (MMR) genes. Diagnosing LS in women with EC can reduce subsequent cancer mortality through colonoscopic surveillance and aspirin chemoprevention; it also enables cascade testing of relatives. A growing consensus supports LS screening in EC; however, the expected proportion of test positives, and optimal testing strategy is uncertain. Previous studies from insurance-based healthcare systems were limited by narrow selection criteria, failure to apply reference standard tests consistently, and poor conversion to definitive testing. The aim of this study was to establish the prevalence of LS and the diagnostic accuracy of LS testing strategies in an unselected EC population.

**Methods and findings:**

This was a prospective cross-sectional study carried out at a large United Kingdom gynaecological cancer centre between October 2015 and January 2017. Women diagnosed with EC or atypical hyperplasia (AH) were offered LS testing. Tumours underwent MMR immunohistochemistry (IHC), microsatellite instability (MSI), and targeted *MLH1*-methylation testing. Women <50 years, with strong family histories and/or indicative tumour molecular features, underwent MMR germline sequencing. Somatic MMR sequencing was performed when indicative molecular features were unexplained by LS or *MLH1*-hypermethylation. The main outcome measures were the prevalence of LS in an unselected EC population and the diagnostic accuracy of clinical and tumour testing strategies for risk stratifying women with EC for MMR germline sequencing. In total, 500 women participated in the study; only 2 (<1%) declined. Germline sequencing was indicated and conducted for 136 and 135 women, respectively. A total of 16/500 women (3.2%, 95% CI 1.8% to 5.1%) had LS, and 11 more (2.2%) had MMR variants of uncertain significance. Restricting testing to age <50 years, indicative family history (revised Bethesda guidelines or Amsterdam II criteria) or endometrioid histology alone would have missed 9/16 (56%), 8/13 (62%) or 9/13 (69%), and 5/16 (31%) cases of LS, respectively. In total 132/500 tumours were MMR deficient by IHC of which 83/132 (63%) had *MLH1*-hypermethylation, and 16/49 (33%) of the remaining patients had LS (16/132 with MMR deficiency, 12%). MMR-IHC with targeted *MLH1*-methylation testing was more discriminatory for LS than MSI with targeted methylation testing, with 100% versus 56.3% (16/16 versus 9/16) sensitivity (*p* = 0.016) and equal 97.5% (468/484) specificity; 64% MSI-H and 73% MMR deficient tumours unexplained by LS or *MLH1-*hypermethylation had somatic MMR mutations. The main limitation of the study was failure to conduct MMR germline sequencing for the whole study population, which means that the sensitivity and specificity of tumour triage strategies for LS detection may be overestimated, although the risk of LS in women with no clinical or tumour predictors is expected to be extremely low.

**Conclusions:**

In this study, we observed that age, family history, and histology are imprecise clinical correlates of LS-EC. IHC outperformed MSI for tumour triage and reliably identified both germline and somatic MMR mutations. The 3.2% proportion of LS-EC is similar to colorectal cancer, supporting unselected screening of EC for LS.

## Abstract 2

Emma Crosbie and colleagues investigate the prevalence of and strategies for identifying Lynch syndrome among women with endometrial cancer.

## Author summary

**Why was this study done?:**

- Endometrial (womb) cancer (EC) is the most common gynaecological cancer in the developed world, and its incidence is rising. A significant minority (around 3%) of EC are caused by an inherited genetic predisposition called Lynch syndrome (LS).
- EC may be the first sign that a woman has LS. She is likely to survive this cancer but develop other preventable cancers related to LS later in life. Her family members are also at risk.
- Identifying women with LS can enable them to reduce their risk of new cancers, for example, through bowel (colorectal) cancer surveillance (colonoscopy).
- We do not know how many women with EC have LS or how best to identify them; current practice is based on experience in bowel cancer and may not be accurate in EC.

**What did this research do and find?:**

- We tested 500 women with EC treated in a large tertiary referral centre in the North West of England for LS.
- We did not preselect women to test based on clinical or tumour characteristics. We tested tumours for features of LS called mismatch repair (MMR) deficiency and microsatellite instability (MSI). Women with strongly suggestive clinical or tumour characteristics underwent germline LS testing.
- In total, 16 of 500 women (3%) had LS, and these women could not always be predicted by their age or family history.
- MMR deficiency was more accurate than MSI at identifying LS-EC, picking up 16/16 (100%) versus 9/16 (56%).

**What do these findings mean?:**

- In our study, we found that 3% of women with endometrial cancer have LS and can benefit from strategies to reduce their future cancer risk.
- Our results suggest that it may be best to test everyone because preselecting women to test based on clinical or tumour characteristics misses cases of LS.
- In this population, tumour MMR deficiency was more accurate than MSI at identifying LS in EC.
- Our results should be interpreted with caution because we did not do germline testing on all women, and the number of women we tested was relatively small.

## Introduction

Endometrial cancer (EC) is the most common gynaecological cancer in developed countries, and incidence is rising [1]. Although mostly driven by obesity and decreased parity, a significant minority is caused by Lynch syndrome (LS), an inherited susceptibility to defective DNA mismatch repair (MMR). At least 1:280 of the general population carries a pathogenic variant in an MMR gene—*MLH1*, *MSH2*, *MSH6* or *PMS2* (*path_MMR*)*—*the vast majority of whom are undiagnosed [2]. The risks of EC, ovarian cancer (OC), and colorectal cancer (CRC) in *path_MMR* heterozygotes are approximately 35%, 11%, and 46%, respectively [3]. These risks are significantly higher than for the general population (EC-3%, OC-1%, and CRC-5.5%) [4].

Often the first malignancy affecting women with LS, EC provides a unique diagnostic opportunity [5]. Most women survive EC [6] but remain at increased risk of associated cancers, particularly CRC [7]. Cascade testing of relatives generates on average 3 further diagnoses per index case [8]. These *path_MMR* carriers can benefit from chemoprophylaxis [9], risk-reducing surgery [10], family planning, and cancer surveillance [3]. Unselected screening of EC for MMR deficiency and/or microsatellite instability-high (MSI-H, a hallmark of MMR deficiency) has advantages that extend beyond LS carrier identification. Programmed cell death protein 1 (PD-1) blockade immunotherapy is most effective in MMR deficient tumours [11], and molecular characterization defines prognosis and treatment eligibility [12].

*Path_MMR* carriers’ association with EC is well established [3]; however, the proportion of EC patients likely to test positive for LS is uncertain, with estimates spanning <1% to >10%. The variation in estimates comes from methodological heterogeneity, small sample sizes, and incomplete testing [13]. Initial tumour triage by immunohistochemistry (IHC) and/or MSI with/without *MLH1*-methylation testing selects women for definitive constitutional analysis [14]. However, the diagnostic accuracy of these strategies is unknown in EC [15]. For instance, MSI testing has reduced sensitivity in *path*_*MSH6* tumours [16]. Selecting test populations by age and/or family history, failure to apply reference standard tests consistently, and poor conversion to definitive testing are all potential sources of bias [13]. Most previous studies involve insurance-based healthcare systems; this is fraught with difficulty because fear of lack of reimbursement by services [17] and increased insurance premiums in individuals [18] influences testing decisions. Thus, the aims of this prospective study were to (1) establish the prevalence of LS and (2) evaluate the diagnostic accuracy of common LS testing strategies in an unselected EC population within a non–insurance-based healthcare system.

## Methods

### Study protocol

The Proportion of Endometrial Tumours Associated with Lynch Syndrome (PETALS) study was sponsored by the University of Manchester, United Kingdom, and approved by the North West Research Ethics Committee (15/NW/0733; S1 Protocol). The study was prospectively registered (Cancer Research-UK clinical trial database, ref-13595). It is reported according to the Strengthening the Reporting of Observational Studies in Epidemiology (STROBE) guideline (S1 Checklist), and the primary data set is also provided (S1 Data).

### Participants

Women were recruited from gynaecology clinics at Manchester University NHS Foundation Trust (MFT), a large gynaecological cancer centre, between October 2015 and January 2017. Women diagnosed with EC or atypical hyperplasia (AH) over the preceding 5 years were eligible for recruitment without demographic or histological restrictions. AH was included to capture the full spectrum of endometrial neoplasia. All women gave written, informed consent to participate, providing blood-DNA, tumour, and clinical data (age, body mass index [BMI], self-reported ethnicity) including detailed family histories (pedigrees). The latter were scored using revised Bethesda [19], Amsterdam II [20], and Prediction of MMR Gene Mutations-v.5 scores (PREMM_5_) [21]. Additional samples were procured from women with EC who had consented to their clinical data, tumours, and DNA being used for future research between 2013 and 2014 at MFT; their detailed pedigrees were not available.

### Somatic tumour analysis

Hysterectomy and biopsy specimens were assessed by 2 specialist gynaecological pathologists according to FIGO-2009 staging criteria (EC) and WHO classification system (AH). Stromal tumour-infiltrating lymphocytes (TILs) were reported as previously described [22]. Tumour molecular profiling used the hysterectomy specimen when possible, but diagnostic endometrial specimens were used when hysterectomy was not performed or when equivocal IHC was repeated. All tissue was formalin-fixed and paraffin-embedded according to local clinical protocols. Tissue blocks with the greatest tumour content (>70%) were chosen for DNA extraction. Tumour was either microdissected from 5 × 10 μm unstained sections or cored from tissue blocks, depending on tumour content. Nonmalignant adjacent tissue was selected for comparative constitutional MSI analysis.

#### IHC

IHC for the 4 MMR proteins was performed using the automated Ventana BenchMark ULTRA IHC⁄in situ hybridisation (ISH) staining module and the OptiView, 3′diaminobenzidine version 5 detection system (Ventana Co., USA) in a laboratory that participates successfully in external quality assurance (EQA; UK NEQAS ICC and ISH, Module 7B; https://www.ukneqasiccish.org; S1 Text). The proportion of stained tumour epithelial component and intensity of staining was scored by 2 expert independent observers using tumour stroma as internal control as previously described [23]. Examples of complete and ‘patchy’ MMR protein loss are illustrated in S1 Text.

#### MSI

MSI (and *MLH1*-methylation) analysis was performed in a UKAS ISO15189-accredited MSI testing reference laboratory that successfully participates in EQA (https://www.genqa.org). DNA was extracted and underwent sodium bisulfite conversion using the Epitect Plus FFPE kit (Qiagen, UK). The MSI analysis system version 1.2 (Promega, USA) was used with standardised clinical protocols. Fluorescent-labelled primers were used to co-amplify 7 markers, including 5 mononucleotide-repeat markers (BAT-25, BAT-26, NR-21, NR-24, and MONO-27), and 2 control pentanucleotide-repeat markers (Penta-C/Penta-D). MSI status was determined by 2 independent scientists. Identical fragment profiles between tumour and matched normal tissue for all 5 mononucleotide loci was considered microsatellite stable (MSS); discordance in one mononucleotide locus was MSI low (MSI-L). Discordance in 2 or more mononucleotide loci was MSI high (MSI-H). Only those with MSI-H tumours were considered at risk of LS; this is consistent with expert consensus [15].

#### Methylation analysis

Reflex *MLH1*-methylation testing was performed on MLH1 and/or PMS2-deficient and/or MSI tumours as previously described [24]. Purified DNA was amplified with bisulfite specific primers in triplicate. An *MLH1* promoter region containing 4 CpG dinucleotides whose methylation status is strongly correlated with *MLH1* expression was analysed using pyrosequencing (PSQ 96MA). Two independent scientists interpreted the pyrograms. ‘Hypermethylation’ described >10% mean methylation across the 4 CpG dinucleotides on over two-thirds of replicate analyses. A proportion of *MLH1*-hypermethylation cases underwent reference standard germline MMR sequencing to exclude co-existing *path*_*MLH1* variants.

### Germline analysis

Indications for germline analysis were age <50 years; meeting revised Bethesda guidelines/Amsterdam II criteria; PREMM_5_ score >10%; and indicative tumour molecular features, specifically MMR deficiency (MMRd, tumour epithelial loss of ≥1 MMR protein on IHC) and/or MSI-H unexplained by somatic *MLH1*-hypermethylation.

DNA was extracted from 2 mL lymphocyte blood (ethylenediaminetetraacetic acid [EDTA] anticoagulant) using Chemagic DNA blood chemistry (CMG-1097-D) on an automated Perkin Elmer Chemagic-360 Magnetic Separation Module and a JANUS Integrator 4-tip Automated Liquid handling platform. DNA was eluted into 400 μL buffer. The concentration and quality of extracted DNA samples were measured using a Nanodrop ND-8000 spectrophotometer. MMR genes *MLH1*, *MSH2*, and *MSH6* were amplified using long-range polymerase chain reaction (PCR) followed by next generation sequencing (NGS) using Illumina SBS version 2 2 × 150 bp and Illumina MiSeq to analyse the coding region, flanking sequences to ±15 bp and known splicing variants (minimum 100× coverage depth) of *MLH1*, *MSH2*, and *MSH6* (S1 Text). Variant identification and calling was via an in-house bioinformatic pipeline. Reported sequence changes and regions with <100× coverage were retested via Sanger sequencing using BigDye version 3.1. Copy number analysis to detect large genomic rearrangements affecting the MMR genes was performed using MLPA MRC-Holland probe mixes: P003-D1 *MLH1*/*MSH2* and P072-C1 *MSH6*. Variant nomenclature followed Human Genome Variation Society (HGVS) guidelines (http://www.hgvs.org/vamomen) using reference sequences: LRG_216,t1(*MLH1*); LRG_218,t1(*MSH2*); LRG_219,t1(*MSH6*). Exons were numbered consecutively starting from exon 1 as the first translated exon for each probe mix. Cases with PMS2 protein loss, normal *MLH1*-methylation, and no *path*_*MLH1/MSH2/MSH6* variant underwent *path*_*PMS2* analysis at the regional specialist Yorkshire and North East Genomic Laboratory. When pathogenicity of the variant was unclear, Ian Frayling was consulted as InSiGHT representative to adjudicate. Somatic MMR sequencing was performed for discordant tumour/germline results (S1 Text).

### Statistical analysis

We determined that a sample size of 497 was required to find a prevalence of LS-EC of 3% (95% confidence intervals 1.5%–4.5%) [25]. The statistical analysis plan was devised *a priori*. Diagnostic accuracy measures were conducted to establish the utility of clinical parameters and tumour triage strategies for risk stratifying women with EC for MMR germline sequencing, including age, family history, histological subtype, density of TILs, MMR deficiency by IHC, MSI status, and *MLH1*-methylation status. There were no data-driven changes to analyses. Descriptive univariate analysis was performed using Student *t* test or two-way ANOVA for normally distributed continuous variables, and Mann–Whitney U test for non-normally distributed continuous variables. Normality of the data was assessed by the Belanger and D’Agostino method with the Royston adjustment (alpha = 0.05) [26]. Pearson’s chi-squared test was used to test for independence of categorical variables. Diagnostic accuracy measures (sensitivity, specificity, and positive and negative predictive values) were calculated using standard formulae, with confidence intervals estimated by the Clopper–Pearson method. The reference standard was germline analysis and only women with *path_MMR* variants (not variants of unknown significance [VUS]) were considered to have LS (disease positive). Women were treated as disease negative (no LS) when germline analysis was not indicated. The exact McNemar’s test was used to compare the sensitivity and specificity of IHC-based versus MSI-based testing for LS identification. Logistic regression was used to identify clinical predictors of MSI, MMR deficiency, *MLH1*-hypermethylation, and germline *path_MMR* variants.

## Results

### Study population

**Fig 1:**
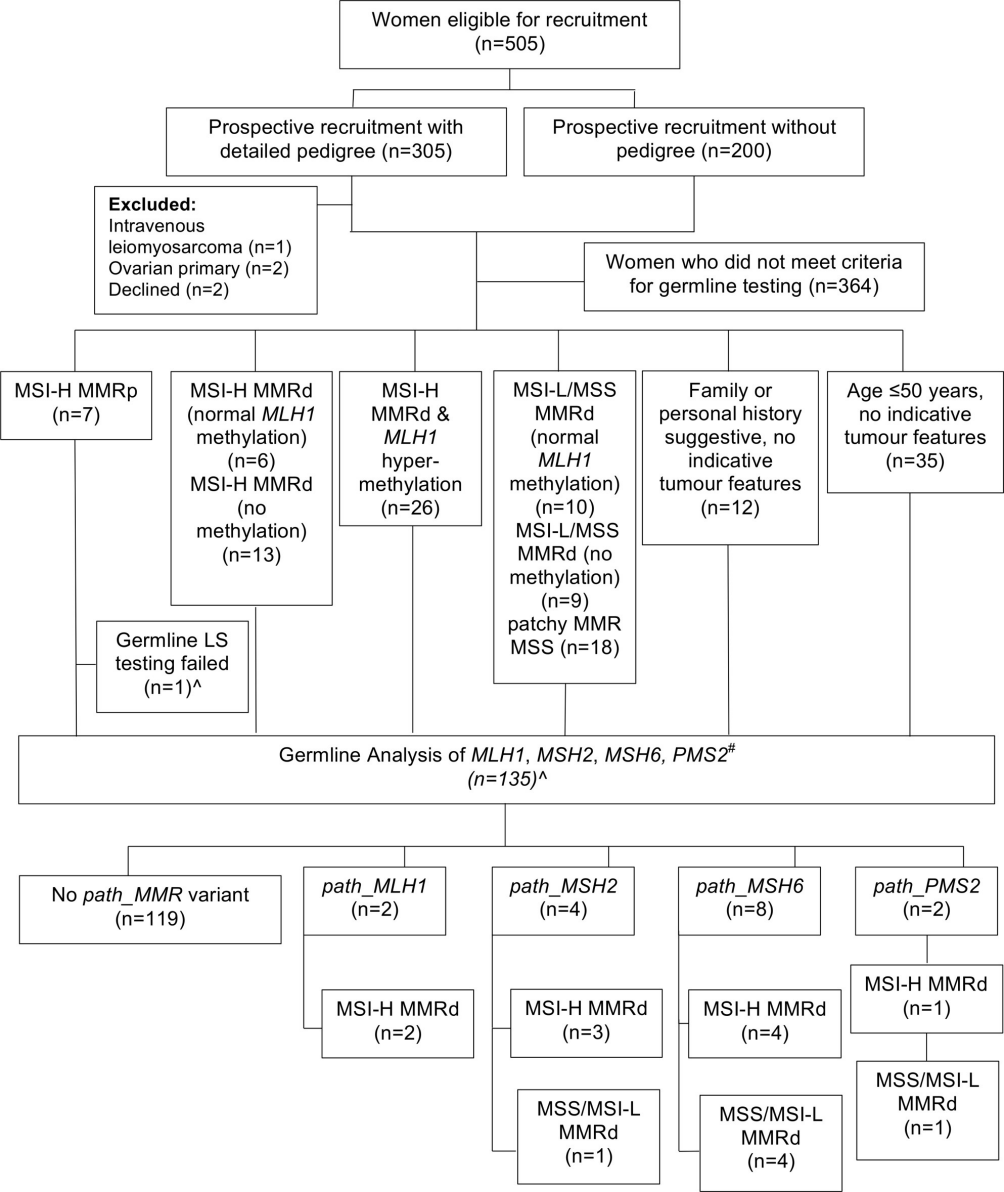
Study flow diagram. Methylation testing only done if ≥1 of MLH1 or PMS2 was lost on immunohistochemistry. “No methylation” denotes it was not indicated. ^#^*PMS2* only tested if PMS2d and no *path*_*MLH1*, *path*_*MSH2* or *path*_*MSH6*. ^One of the MSI-H samples did not undergo germline testing as the patient died before blood could be taken. MSI, microsatellite instability; MSS, microsatellite stable; MMR, mismatch repair; MMRp, MMR proficient (no MMR protein loss); MMRd, MMR deficient (≥1 MMR protein lost).

In total, 305 women were invited to participate in PETALS and undergo testing for LS (pedigree cohort). Only 2 declined, and 3 were ineligible (not EC/AH on final pathology; Fig 1). A further 200 women treated for AH/EC at MFT in the preceding 2 years (2013–2014) were included, but detailed family histories were unavailable (nonpedigree cohort). The final study population comprised 500 women with median age and BMI of 65-years and 32kg/m^2^, respectively, of predominantly white British ethnicity (81%; Table 1). There were 470 EC cases (94%) and 30 AH (6%). Most EC were low grade (62%) and early stage tumours (72%) of endometrioid subtype (70%). All 500 women underwent both MMR-IHC and MSI analysis, with targeted *MLH1-*methylation testing. Of these, 135 women underwent germline LS testing for the following indications: MSI-H MMR deficient tumour with normal *MLH1-*methylation (*n* = 6); MSI-H MMR deficient tumour (*MLH1*-methylation testing not indicated; *n* = 13); MMR deficient and MSS/MSI-L (*n* = 19); MSI-H MMR-proficient tumour (*n* = 6); age ≤50 years (n = 35); and strong family history (n = 12). A subset of women with tumour *MLH1*-hypermethylation (*n* = 26) and subset of MSS/MSI-L patchy tumour MMR deficiency (*n* = 18) also underwent testing (see Fig 1).

**Table 1:** Patient demographics and clinical characteristics.

Patient characteristics	Overall (*n* = 500)	Pedigree cohort (*n* = 300)	Nonpedigree cohort (*n* = 200)	*P* value^a^
**Age, years [median (IQR)]**	**65 (56–73)**	**65 (56–73)**	**65 (56–72.5)**	**0.72**^**b**^
>80	30 (6.0%)	15 (5.0%)	15 (7.5%)	
60–80	295 (59.0%)	179 (59.7%)	116 (58.0%)	
51–59	102 (20.4%)	63 (21.0%)	39 (19.5%)	
≤50	73 (14.6%)	43 (14.3%)	30 (15.0%)	
**Ethnicity**				**0.055**^**c**^
White	405 (81.0%)	248 (82.7%)	157 (78.5%)	
Black	20 (4.0%)	10 (3.3%)	10 (5.0%)	
Asian	55 (11.0%)	30 (10.0%)	25 (12.5%)	
Chinese	10 (2.0%)	3 (1.0%)	7 (3.5%)	
Other	10 (2.0%)	9 (3.0%)	1 (0.5%)	
**BMI, kg/m**^**2**^ **[median, range]**	**31.6 (16.6–71.0)**	**31.3 (16.8–69.5)**	**32.0 (16.6–71.0)**	**0.11**^**b**^
Underweight [0–18.5]	5 (1.0%)	2 (0.7%)	3 (1.5%)	
Normal [18.5–25]	89 (17.9%)	62 (20.7%)	27 (13.6%)	
Overweight [25–30]	109 (21.9%)	62 (20.7%)	47 (23.7%)	
Obese Class I [30–35]	111 (22.3%)	69 (23.0%)	42 (21.2%)	
Obese Class II [35–40]	67 (13.5%)	44 (14.7%)	23 (11.6%)	
Obese Class III [40–45]	46 (9.2%)	23 (7.7%)	23 (11.6%)	
Obese Class IV [45–50]	25 (5.0%)	17 (5.7%)	8 (4.0%)	
Obese Class V [50–60]	34 (6.8%)	17 (5.7%)	17 (8.6%)	
Obese Class VI [≥60]	12 (2.4%)	3 (1.3%)	8 (4.0%)	
**Grade of tumour**				**0.001**^c^*
Atypical hyperplasia	30 (6.0%)	8 (2.7%)	22 (11.0%)	
1	209 (41.8%)	134 (44.7%)	75 (37.5%)	
2	101 (20.2%)	58 (19.3%)	43 (21.5%)	
3	160 (32.0%)	100 (33.3%)	60 (30.0%)	
**FIGO (2009) stage**				**<0.001**^c^*
Atypical hyperplasia	30 (6.0%)	8 (2.7%)	22 (11.0%)	
I	360 (72.0%)	221 (73.7%)	139 (69.5%)	
II	48 (9.6%)	37 (12.3%)	11 (5.5%)	
III	59 (11.8%)	32 (10.7%)	27 (13.5%)	
IV	3 (0.6%)	2 (0.7%)	1 (0.5%)	
**Histological subtype**				**0.001**^c^*
Atypical hyperplasia	30 (6.0%)	8 (2.7%)	22 (11.0%)	
Endometrioid	351 (70.2%)	214 (71.3%)	137 (68.5%)	
Serous	33 (6.6%)	28 (9.3%)	5 (2.5%)	
Clear cell	23 (4.6%)	13 (4.3%)	10 (5.0%)	
Carcinosarcoma	34 (6.8%)	21 (7.0%)	13 (6.5%)	
Dedifferentiated	10 (2.0%)	6 (2.0%)	4 (2.0%)	
Mixed	19 (3.8%)	10 (3.3%)	9 (4.5%)	
**Sample type**				**0.017**^c^*
Biopsy	44 (8.8%)	19 (6.3%)	25 (12.5%)	
Hysterectomy	456 (91.2%)	281 (93.7%)	175 (87.5%)	

^a^*P* value compares pedigree and nonpedigree cohorts.

^b^*P* value from Mann–Whitney U test.

^c^*P* value from Pearson’s χ^2^ test.

*Denotes statistical significance at alpha = 0.05.

**Abbreviations:** BMI, body mass index; FIGO, International Federation of Gynecology and Obstetrics; IQR, interquartile range

### Proportion of tumours associated with LS

We identified 16 *path_MMR* variant carriers, giving a 3.2% overall LS prevalence (95% CI 1.84%–5.14%). There were 8 *path_MSH6*, 4 *path_MSH2*, 2 *path_MLH1*, and 2 *path_PMS2* variant carriers (Table 2). Three had known LS, 2 died shortly after EC diagnosis, and the remaining 11 were offered, of whom 10 accepted, genetic counselling. To date, 14 relatives have also received genetic counselling. One woman carried 2 *VUS*_*MSH6*, considered pathogenic in combination (S2 Text). A further 10 women had MMR variants that were not recognized by InSiGHT (https://www.insight-group.org), including 5 previously unreported variants (S3 Text). Greatest discrepancy between IHC and MSI findings was observed for those with a germline path_*MSH6* variant with 5/8 demonstrating MSH6 loss on IHC but MSS. One path_*MSH6* variant (*MSH6* c.2731C>T p.(Arg911Ter) was observed in 3 index cases and therefore could represent a local founder mutation; however, review of the local clinical database indicates this variant only affects 5/487 of local LS families.

**Table 2:** Clinicopathological characteristics, tumour-based triage, and germline sequencing results of women with LS-associated EC.

Patient	Demographics			Family history			Pathology	Tumour-based triage		Germline sequencing		
ID	Age range (y)	BMI (kg/m^2)^	Ethnicity	Meets Amsterdam II Criteria	Meets Revised Bethesda Criteria	PREMM_5_ score	FIGO (2009) stage, grade and histological subtype	MMR-IHC results	MSI results	Germline pathological variant	InSiGHT class	ACMG class
16^a^	30–34	23	White	Yes	Yes	17.80%	Stage 1a grade 3 mixed endometrioid and clear cell EC	MLH1/PMS2 loss (normal *MLH1*-methylation)	MSI-H	*MLH1* c.473delA p.(Asn158ThrfsTer2)	5*	P
25	50–54	30	White	Yes	Yes	7.60%	Stage 3b grade 2 endometrioid EC	MSH2/MSH6 loss	MSI-H	*MSH2* c.2563C>T p.(Gln855Ter)	5	P
31^a^	40–44	25	White	No	No	9.10%	Stage 1a grade 1 endometrioid EC	Isolated MSH6 loss	MSS	*MSH6* c.2731C>T p.(Arg911Ter)	5	P
61	45–49	42	Asian	Yes	Yes	24.20%	Stage 3a grade 3 de-differentiated EC	MSH2/MSH6 loss	MSI-H	*MSH2* Ex n7 deletion	5	P
96	80–84	29	White	No	No	2.00%	Stage 3b grade 2 endometrioid EC	Isolated MSH6 loss	MSS	*MSH6* c.1084C>T p.(Pro362Ser) & *MSH6* c.2018C>T p.(Pro673Leu)	4*	VUS
122	45–49	20	White	No	No	11.30%	Stage 1a grade 1 endometrioid EC	MSH2/MSH6 loss	MSI-H	*MSH2* c.366+1G>A	4	P
128	60–64	23	White	No	No	2.50%	Stage 1b grade 1 endometrioid EC	Isolated MSH6 loss	MSI-L	*MSH6* c.3313G>T p.(Gly1105Ter)	5*	LP
173	65–69	23	White	No	No	2.20%	Stage 1a grade 2 endometrioid EC	PMS2 loss	MSI-H	*PMS2* Del Exon 9–10	5	P
213	44–49	27	White	No	No	4.80%	Stage 2 grade 3 mixed endometrioid and clear cell EC	Isolated MSH6 loss	MSI-H	*MSH6* c.2731C>T p.(Arg911Ter)	5	P
215	60–64	33	White	No	No	2.50%	Stage 3b grade 2 endometrioid EC	Isolated MSH6 loss	MSI-H	*MSH6* c.3004_3005delGG p.(Gly1002LeufsTer2)	5*	P
241	60–64	36	White	No	No	3.10%	Stage 1b grade 3 carcinosarcoma	Isolated MSH6 loss	MSI-H	*MSH6* c.2731C>T p.(Arg911Ter)	5	P
255	55–59	34	White	No	No	6.40%	Stage 1a grade 3 endometrioid EC	Isolated MSH6 loss	MSS	*MSH6* c.2731C>T p.(Arg911Ter)	5	P
256^a^^,^^b^	45–49	32	White	Yes	Yes	8.60%	Stage 1a grade 1 endometrioid EC	MLH1/PMS2 loss (normal *MLH1*-methylation)	MSI-H	*MLH1* c.1409+1 G>C	5	P
BRC 882	25–29	21	Asian	No	Yes	27.20%	Stage 1a grade 1 endometrioid EC	Isolated PMS2 loss	MSS	Homozygous *PMS2* c.1500delC	5*	P
BRC 165	65–69	23	White	No	No	3.00%	Stage 3a grade 3 carcinosarcoma	MSH2/MSH6 loss	MSS	*MSH2* Del Exon 1–8	5	P
PRE 011	55–59	30	White	No	No	3%	Stage 1a grade 1 endometrioid EC	Isolated MSH6 loss	MSS	*MSH6* c.3261delC p.(Phe1088SerfsTer2)	5	P

^a^Already aware of LS diagnosis before enrolment in PETALS study.

^b^Enrolled in gynaecological cancer surveillance program, EC incidental finding at risk reducing prophylactic hysterectomy.

InSiGHT: class 5, pathogenic MMR variant; class 4, likely pathogenic MMR variant; class 3, MMR variant of uncertain pathogenicity.

ACMG classification of MMR variants: P, pathogenic; LP, likely pathogenic; VUS, variant of uncertain significance.

**Abbreviations:** ACMG, American College of Medical Genetics and Genomics; BMI, body mass index; EC, endometrial cancer; FIGO,; IHC, immunohistochemistry; InSiGHT,; LS, Lynch Syndrome; MMR, mismatch repair; MSI, microsatellite instability; MSI-H, microsatellite instability-high; MSI-L, microsatellite instability-low; MSS, microsatellite stable; PREMM_5_, Prediction of MMR Gene Mutations-v.5 scores

### Selecting women for germline LS testing

#### Age and family history

In total, there were 73 women ≤50 years, of whom 7 (10%) had LS. All 7 had indicative tumour molecular features (MMRd ± MSI-H). Only screening women <50, <60, and <70 years would have missed 9, 6, and one LS diagnosis, respectively (Table 3). A further 35 women <50 years with MSS/MSI-L MMR-proficient tumours underwent germline testing for LS; 4 (11.4%) had *VUS_MMR*, but no further *path_MMR* variants were identified.

**Table 3:** Diagnostic test accuracy of clinicopathological selection criteria and tumour-based triage strategies.

Clinicopathological variable	Sensitivity (%)	Specificity (%)	PPV (%)	NPV (%)
**MMR deficiency by IHC**	100 (79.4–100)	80.6 (76.8–84.0)	14.5 (8.5–22.5)	100 (99.1–100)
With *MLH1*-methylation testing	100 (79.4–100)	96.7 (94.7–98.1)	50.0 (31.9–68.1)	100 (99.2–100)
**MSI-H**	56.3 (29.9–80.2)	83.5 (79.9–86.7)	10.1 (4.7–18.3)	98.3 (96.5–99.3)
With *MLH1*-methylation testing	56.3 (29.9–80.2)	96.7 (94.7–98.1)	36.0 (18.0–57.5)	98.5 (97.0–99.4)
**MMR deficiency or MSI-H**	100 (79.4–100)	79.1 (75.2–82.7)	13.7 (8.0–21.3)	100 (99.0–100)
With *MLH1*-methylation testing	100 (79.4–100)	95.5 (93.2–97.1)	42.1 (26.3–59.2)	100 (99.2–100)
**Age**				
<50 years	43.8 (19.8–70.1)	87.4 (84.1–90.2)	10.3 (4.2–20.1)	97.9 (96.1–99.0)
<60 years	56.3 (29.9–80.2)	65.7 (61.3–69.9)	5.1 (2.4–9.5)	97.8 (95.6–99.1)
<70 years	93.8 (69.8–99.8)	35.1 (30.9–39.6)	4.6 (2.6–7.4)	99.4 (96.8–100.0)
**BMI <35 kg/m**^**2**^	86.7 (59.5–98.3)	37.7 (33.3–42.2)	4.1 (2.2–7.0)	98.9 (96.1–99.9)
<50 years	33.3 (11.8–61.6)	94.8 (92.5–96.6)	16.7 (5.6–34.7)	97.9 (96.1–99.0)
<60 years	46.7 (21.3–73.4)	82.0 (78.3–85.3)	7.4 (3.0–14.7)	98.0 (96.1–99.1)
<70 years	80.0 (51.9–95.7)	64.0 (59.5–68.3)	6.5 (3.4–11.0)	99.0 (97.2–99.8)
**PREMM**_**5**_ **score**	84.6 (54.6–98.1)	46.5 (40.6–52.5)	6.7 (3.4–11.7)	98.5 (94.8–99.8)
**Amsterdam II Criteria**	30.8 (9.1–61.4)	99.0 (97.0–99.8)	57.1 (18.4–90.1)	96.9 (94.2–98.6)
**Revised Bethesda Guidelines**	38.5 (13.9–68.4)	98.6 (96.5–99.6)	55.6 (21.2–86.3)	97.3 (94.7–98.8)
**Endometrioid histopathology**	68.8 (41.3–88.9)	29.8 (25.7–34.0	3.1 (1.6–5.5)	96.6 (92.3–98.9)
**High TILs**	93.8 (69.8–99.8)	76.7 (72.6–80.4)	11.7 (6.7–18.6)	99.7 (98.5–100)

**Abbreviations:** BMI, body mass index; IHC, immunohistochemistry; MMR, mismatch repair; MSI, microsatellite instability; MSI-H, microsatellite instability-high; NPV, negative predictive value; PPV, positive predictive value; PREMM, PREdiction Model for gene Mutations; TIL, tumour-infiltrating lymphocyte

Comprehensive pedigree data were available for 300 women. Only 7/300 (2%) and 9/300 (3%) women with detailed pedigrees met the Amsterdam II criteria and revised Bethesda guidelines, of whom 4 (57%) and 5 (56%) had LS, respectively. All women with LS also had indicative tumour molecular features. The overall mean PREMM_5_ score was 3.2% (SD 2.4%). A total of 164/299 (55%) women had scores greater than the 2.5% recommended cut-off for germline testing, with mean PREMM_5_ score 4.3% in this subgroup, and 11 (6.7%) having LS. An additional 12 women with MSS/MSI-L MMR-proficient tumours underwent germline testing because of a previous LS-associated cancer (*n* = 3) or an indicative family history (including Amsterdam II [*n* = 2], revised Bethesda [*n* = 4], and PREMM_5_ > 10% [*n* = 3]). None carried a *path_MMR* variant or *VUS_MMR*.

#### MMR deficiency by IHC

In total 132/500 (26%) tumours were MMR deficient (Table 4), of which 83 were MSI-H. Women with MMR deficient tumours were older than those without MMR loss (mean difference 3.3 years, *t* test [unequal variances] *p* = 0.007). Of the 24 women who had tumours with patchy MMR loss, one had a germline *VUS_MMR* but none had LS. One MSS AH case had patchy MSH6 loss and was subsequently found to have a VUS_*MSH6*, but all other AH samples had intact MMR. Thirteen tumours failed first attempt but not repeat MMR-IHC testing.

**Table 4:** Molecular analysis of MMR deficient, *MLH1*-hypermethylated, and MSI-H tumours.

Tumour characteristic		*n*	MSI-H	MSI-L	*MLH1*-hypermethylated	*path_MMR* variant	*VUS_MMR*	Somatic MMR mutation	Unexplained when all tests completed	% unexplained
**MMR proficient**		368	7^a^	18	N/A	0	4	N/A	N/A	N/A
**MMR deficient**		132^b^	81	3	82^e^	16	7	16^c^^,^^d^	16/127^f^^,^^g^	12.6%
	**Complete MMR deficiency**									
	Overall	108^b^	80	2	77	16	6	12^c^	2/106^f^	1.9%
	MLH1 only	2	2	0	2	0	0	N/A	0/2	0%
	MLH1 and PMS2	82	65	2	75	2	2	2	0/80^f^	0%
	PMS2 only	2	0	0	0	2	0	N/A	0/2	0%
	MSH2 only	0	0	0	0	0	0	N/A	N/A	N/A
	MSH2 and MSH6	9	7	0	0	4	1	4	1/9	11%
	MSH6 only	12	5	0	0	8	2	6	1/12	8.5%
	MLH1 and PMS2 and MSH6	1	1	0	1	0	1	0	0/1	0%
	**Patchy MMR deficiency**									
	Overall	24	1	1	5^e^	0	1	4^d^	14/21^g^	67%
	MLH1 only	4	1	0	2	0	0	0	2/4	50%
	MLH1 and PMS2	13	0	1	3	0	0	1	6/10^g^	60%
	PMS2 only	2	0	0	0	0	0	0	2/2	100%
	MSH2 only	1	0	0	0	0	0	1	0/1	N/A
	MSH2 and MSH6	2	0	0	0	0	0	2	2/2	100%
	MSH6 only	2	0	0	0	0	1	0	1/2	50%
***MLH1*-hypermethylated**		83^e^	64	1	0	0	0			
	**Complete MMR deficiency**									
	Overall	78	63	1	N/A	0	0			
	MLH1 only	2	2	0	N/A	0	0			
	MLH1 and PMS2	75	61	1	N/A	0	0			
	PMS2 only	1	0	0	N/A	0	0			
	MLH1 and PMS2 and MSH6	1	1	0	N/A	0	0			
	**Patchy MMR deficiency**									
	Overall	5^e^	1	0	N/A	0	0			
	MLH1 only	2	1	0	N/A	0	0			
	MLH1 and PMS2	3	0	0	N/A	0	0			
	PMS2 only	0	0	0	N/A	0	0			
**Normal *MLH1*-methylation**		16^h^	6	1	N/A	3	3			
	**Complete MMR deficiency**									
	Overall	9	6	0	N/A	3	2			
	MLH1 only	0	0	0	N/A	0	0			
	MLH1 and PMS2	8	6	0	N/A	2	2			
	PMS2 only	1	0	0	N/A	1	0			
	MLH1 and PMS2 and MSH6	0	0	0	N/A	0	0			
	**Patchy MMR deficiency**									
	Overall	6	0	1	N/A	0	1			
	MLH1 only	1	0	0	N/A	0	0			
	MLH1 and PMS2	4	0	1	N/A	0	1			
	PMS2 only	1	0	0	N/A	0	0			

^a^One of the MSI-H samples did not undergo germline testing as the patient died before blood could be taken.

^b^One sample defined as inconclusive IHC loss after MDT review.

^c^Includes one mono-allelic without loss of heterogeneity VUS.

^d^Includes 2 mono-allelic without loss of heterogeneity VUS.

^e^One sample failed methylation analysis multiple times.

^f^Two complete MMRd samples failed somatic analysis (both MLH1/PMS2 loss).

^g^Three ‘patchy’ MMRd samples failed somatic analysis (all MLH1/PMS2 IHC loss).

^h^One sample (ID R125) underwent *MLH1*-methylation analysis on the initial interpretation of IHC loss in MLH1; this was then revised by the MDT to no loss.

**Abbreviations:** IHC, immunohistochemistry; MDT, multidisciplinary team; MMR, mismatch repair; MMRd, mismatch repair deficient on IHC; MSI-H, microsatellite instability-high; MSI-L, microsatellite instability-low; N/A, not applicable; VUS, variant of unknown significance

#### MSI analysis

In total, 89/500 (18%) tumours were MSI-H, and 21/500 (4%) were MSI-L. None of the 6 MSI-H MMR-proficient tumours were LS-associated (Table 4). Women with MSI-H tumours were older than those with MSS tumours (mean difference 4.7 years, *p* < 0.001). Mononucleotides BAT-25 and/or BAT-26 accounted for 95% of MSI-H events. No AH samples were MSI-H. Eights tumours failed first attempt but not repeat MSI analysis.

#### *MLH1*-methylation analysis

In total, 100 tumours underwent reflex *MLH1*-methylation analysis because of MLH1-deficiency on IHC. Of these, 83 (16% of 500) were hypermethylated (Table 4), and none of the 26 hypermethylated cases (31%) who underwent germline analysis had LS. One sample failed *MLH1*-methylation analysis several times. Women with *MLH1*-hypermethylated tumours were older than those with MMR-proficient tumours (*t* test [unequal variance] *p* < 0.001) and those with normal *MLH1-*methylation MMR deficient tumours (*t* test [unequal variance] *p* = 0.0034). Of 15 women with normal *MLH1*-methylation MMR deficient tumours, 3 (20%) had LS, all of which had complete IHC loss (3/10, 30%).

### Somatic MMR sequencing

Somatic MMR testing was performed when indicative tumour molecular features were unexplained by LS or *MLH1*-hypermethylation: MSI-H MMR deficient (*n* = 8), MSS/MSI-L MMR deficient (*n* = 7), MSI-H MMR-proficient (*n* = 6), patchy MMR deficient (*n* = 18), and germline *VUS_MMR* (*n* = 1) cases (S4 Text). Six tumours failed (MSI-H MMR deficient [*n* = 2], MSI-H MMR-proficient [*n* = 1], and patchy MMR deficient [*n* = 3] cases). This comprehensive testing left just 1.9% (2/106) MMR deficient tumours unexplained by a *path_MMR* variant/epigenetic silencing (Table 4).

### Diagnostic test accuracy of tumour triage

MMR-IHC was superior to MSI analysis for the identification of LS (Table 3). Sensitivity and specificity were 100% versus 56.3% (16/16 versus 9/16; difference 43.75%, 95% CI 13.2%–74.3%, *p* = 0.016) and 80.6% versus 83.5% (390/484 versus 404/484; difference −2.9%, 95% CI −5.2% to −0.6%, *p* = 0.013), respectively. Specificity was increased to 97.7% for both MMR-IHC and MSI with reflex *MLH1*-methylation testing (*p* = 1). The area under the ROC curve for PREMM_5_ and age was 0.73 versus 0.71, respectively, with PREMM_5_ superior to age, although neither matched MMR-IHC for selecting women for germline LS testing (S5 Text). Eleven LS-associated tumours (69%) were of pure endometrioid histotype, and 15 (94%) had high tumour-infiltrating lymphocyte counts. MMR germline sequencing was conducted for 135/500 women, which means that the sensitivity and specificity of tumour triage strategies for LS detection may be overestimated in this study, although the risk of LS in women with no clinical or tumour predictors is expected to be extremely low.

### Clinical predictors of test outcomes

Increased age was positively associated with tumour MMR deficiency (OR 1.02, 95% CI 1.00–1.04, *p* = 0.013), MSI-H (OR 1.03, 95% CI 1.01–1.05, *p* = 0.003), and *MLH1*-hypermethylation (OR 1.04, 95% CI 1.02–1.06, *p* < 0.001) (tumours in which methylation testing was not indicated were assumed to have normal *MLH1* methylation), whilst negatively associated with LS (0.95, 95% CI 0.92–0.99, *p* = 0.006). PREMM_5_ was positively associated with LS (OR 3.88, 95% CI 1.74–8.65, *p* = 0.001) but not with tumour test outcomes (IHC, MSI, or *MLH1*-hypermethylation). BMI was negatively associated with MMR deficiency (OR 0.98, 95% CI 0.96–1.00, *p* = 0.036) and marginally negatively associated with LS (OR 0.93, 95% CI 0.87–1.00, *p* = 0.055). Smoking was not consistently associated with any test outcomes.

## Discussion

In this prospective study, we found a 3.2% prevalence of LS in an unselected EC population and established MMR-IHC with targeted *MLH1*-methylation testing as the most accurate method of selecting women for germline LS testing. MSI was insufficiently sensitive, missing nearly half of all LS-EC. Family history by PREMM_5_ score showed excellent sensitivity but poor specificity; the reverse was true for Amsterdam II criteria/revised Bethesda guidelines. Restricting testing to women <60 years would miss a third of all LS cases, but only one case was >70 years.

The 3.2% prevalence of LS-EC is consistent with the results of our recent systematic review [13]. Only 6 previous studies tested unselected EC populations of ≥500 women for LS, all of which were conducted in the insurance-based healthcare systems of the US and Australia [27–32]. This impacted both the proportion of eligible women consenting to study participation as well as their willingness to undergo definitive germline testing [17]. To our knowledge, this is the first unselected EC population-based study of LS testing conducted in the fully state-funded healthcare system of the UK. We recruited >99% of newly diagnosed EC patients attending our institution during the recruitment period, all tumours underwent both IHC and MSI analysis, and germline LS testing was conducted for 135/136 eligible women. We found MSI analysis had a poor sensitivity, most notably in *path*_*MSH6* carriers. This phenomenon has been described previously [16]; however, no large EC studies to date have germline tested all women with both MMR deficient and MSI-H tumours to enable a direct comparison between the 2 tumour triage strategies [13]. For example, Goodfellow and colleagues carried out germline LS testing on just 5% of their population-based cohort, including those with MSH6-deficient MSI-H tumours (15/107 MMR deficient tumours with normal *MLH1* methylation) but not MSH6-deficient MSI-L or MSS tumours (6/107); it is therefore not possible to calculate the sensitivity of MSI analysis for *path*_*MSH6* carriers from these data [29]. Hampel and colleagues found all 6 endometrial tumours from *path*_*MSH6* carriers were MMR deficient, but just 3/6 were MSI-H; despite this, tumours were triaged for MMR germline sequencing using the results of MSI analysis, and only a subset underwent MMR-IHC [8]. Our comprehensive testing strategy also identified somatic *path_MMR* variants in MMR deficient and/or MSI-H tumours unexplained by LS. This had been noted to a lesser extent in previous studies [33,34] and is an important finding because it removes many of the tumours that would otherwise have been considered ‘Lynch-like’ and posed a clinical management dilemma [35].

There were 5 key strengths to our study. First, we recruited 99% of eligible women, ensuring an unbiased population of consecutive patients unrestricted by age, histological subtype, or treatment modality. Recruitment rates of around 50% have been reported previously, mainly from insurance-based healthcare systems [28]. Second, all tumours underwent MMR-IHC, MSI, and targeted *MLH1*-methylation testing, and all but one woman with indicative tumour features underwent germline *path_MMR* testing. This compares favourably to most studies with incomplete testing [13] and allowed us to test selection criteria and tumour-based triage strategies. Third, all analyses were carried out to quality-assured clinical standards in specialist pathology and genetics referral laboratories. Fourth, all but 2 of the 106 (1.9%) MMR deficient tumours were explained by *MLH1*-hypermethylation, somatic MMR mutation, or LS. Fifth, our inclusion of AH, part of the spectrum of LS-EC [36], is unusual but justified because identifying LS in women with AH not only supports future risk-reducing interventions but also impacts their immediate treatment decisions.

The main limitation was failure to conduct germline LS sequencing on the whole study population. In total, 135/500 (27%) women underwent germline testing, including every woman with established clinical (age ≤50 years, positive family history) or tumour characteristics (MMR deficient or MSI-H unexplained by *MLH1*-hypermethylation) predictive of LS; thus the prevalence of LS in the 365 (73%) women who did not undergo germline LS sequencing is expected to be extremely low. Further, our 27% germline sequencing rate is considerably higher than that reported by other population-based studies in EC [13]. Other limitations include the small sample size with concomitantly few LS diagnoses and correspondingly wide CIs around our sensitivity and specificity estimates. Nevertheless, our study was large enough to detect a significant difference in sensitivity between MMR deficiency and MSI-based tumour triage. All women were treated at one gynaecological cancer centre in North West England; although this might hinder generalizability to other populations, the clinicopathological characteristics of our EC population reflect the UK national picture. The relatively high proportion of *path_MSH6* variant carriers in our cohort affected the sensitivity of MSI-based tumour triage, and our study may underestimate its true value within a wider geographical distribution, although 2 women with *path*_*MSH2* and homozygous *path*_*PMS2* variants also demonstrated MSS tumour phenotypes. Pedigree data were not available for all patients and were self-reported and therefore prone to error, mirroring the situation in routine clinical practice.

Our findings support the unselected screening of EC for LS. We show that age, family history, and pathological findings are of limited value in selecting women for testing. Universal germline testing is not financially feasible [37], mandating tumour-based triage. How best to achieve this is contentious, but the prevailing wisdom is that MSI and IHC are equivalent [29], despite limited evidence to support this [15]. Our data provide strong evidence that MMR-IHC with targeted *MLH1*-methylation testing is superior to MSI-based testing in EC. It reduces the proportion of women requiring germline sequencing without missing any cases, lowering costs [37], and improving cost-effectiveness [38, 39]. The identification of MMR deficient EC is of clinical importance. It allows clinicians to tailor treatments [40], explain prognosis [40], predict cancer recurrence [41], and individualize follow-up [41]. Furthermore, women want to be tested; it is striking that 99% of eligible women approached during routine gynaecological cancer care agreed to participate, and 10/11 newly identified LS carriers attended genetic counselling and supported cascade testing of at-risk family members.

Unselected screening of EC for LS leads to the discovery of VUS_MMR. These create a clinical conundrum because new variants can be challenging to classify. Indeed, 15 of our 27 MMR variants were not previously reported to either the ClinVar or InSiGHT data sets. This highlights the need for international multidisciplinary expert teams to explore the clinical significance of VUS_MMR and/or investment in saturation genome editing platforms for high throughput analysis [15]. Such infrastructure is established for MMR variant interpretation and behoves all clinical laboratories to interpret variants according to a single set of defined criteria (https://www.insight-group.org/criteria/) before universal screening for LS in EC begins in earnest.

To our knowledge, this is the most comprehensive study to date exploring the prevalence of LS in an unselected EC population treated within a non–insurance-based healthcare system. Consent for LS testing was taken by gynaecologists during routine clinical care [42]. In this cohort, we found IHC outperforms MSI as a means of tumour-based triage and reliably identifies both germline and somatic MMR deficient tumours to inform clinical care. The overall prevalence of LS in EC was 3.2%, which is comparable to that of CRC [30], and justifies a similar recommendation for unselected LS screening. We endorse, when resources allow, the universal screening of EC for LS using IHC, targeted *MLH1*-methylation testing, and, where indicated, germline sequencing for *path_MMR* variants.

## Supporting information

S1 Checklist STROBE Checklist. (DOCX)

S1 Protocol PETALS Study Protocol. PETALS, Proportion of Endometrial Tumours Associated with Lynch Syndrome.
(PDF)

S1 Data PETALS primary data file. PETALS, Proportion of Endometrial Tumours Associated with Lynch Syndrome.
(XLSX)

S1 Text Extended immunohistochemistry, germline, and somatic sequencing methods. (DOCX)

S2 Text Reason for mutational analysis and mutation classification in unclear or unknown MMR mutations. MMR, mismatch repair.
(DOCX)

S3 Text Clinical details of carriers of germline MMR variants of uncertain significance. MMR, mismatch repair.
(DOCX)

S4 Text Clinical details of carriers of somatic MMR mutations. MMR, mismatch repair.
(DOCX)

S5 Text Sensitivity and specificity analyses of tumour triage strategies for germline Lynch syndrome testing in endometrial cancer. (DOCX)

## Abbreviations

AH: atypical hyperplasia
BMI: body mass index
CRC: colorectal cancer
EC: endometrial cancer
EDTA: ethylenediaminetetraacetic acid
EQA: external quality assurance
FIGO: International Federation of Gynecology and Obstetrics
HGVS: Human Genome Variation Society
IHC: immunohistochemistry
ISH: in situ hybridization
LS: Lynch syndrome
MDT: multidisciplinary team
MFT: Manchester University NHS Foundation Trust
MMR: mismatch repair
MMRd: MMR deficiency
MMS: microsatellite stable
MSI: microsatellite instability
MSI-H: MSI high
MSI-L: MSI low
NGS: next generation sequencing
OC: ovarian cancer
PCR: polymerase chain reaction
PD-1: programmed cell death protein 1
PETALS: Proportion of Endometrial Tumours Associated with Lynch Syndrome
PREMM_5_: Prediction of MMR Gene Mutations-v.5 scores
TIL: tumour-infiltrating lymphocyte
VUS: variants of unknown significance
