# Supplementary material for: The proportion of endometrial tumours associated with Lynch syndrome (PETALS): A prospective cross-sectional study
Source: PLoS Med. 2020 Sep 17;17(9):e1003263. doi: 10.1371/journal.pmed.1003263 (PMC7497985; doi:10.1371/journal.pmed.1003263)
Supplement: S1 Protocol — PETALS, Proportion of Endometrial Tumours Associated with Lynch Syndrome. (PDF) [file pmed.1003263.s002.pdf]

Proportion of Endometrial Tumours Associated with Lynch Syndrome (PETALS) Study Protocol

Version: 1.0
Date: July 2015
Sponsor: Central Manchester University Hospitals NHS Foundation Trust (CMFT)

Abbreviations and Glossary

**EC**	Endometrial Cancer
**LS**	Lynch Syndrome (Also known as Hereditary non-polyposis colon cancer)
**NGS**	Next Generation Sequencing
**LVSI**	Lymphovascular space involvement
**LN**	Lymph Node
**IHC**	Immunohistochemistry
**MMR**	Mismatch repair
**MLH1**	MutL homolog 1
**MSH2**	MutS protein homolog 2
**MSH6**	MutS protein homolog 6
**PMS2**	postmeiotic segregation increased 2
**MSI**	Microsatellite instability
**CRC**	Colorectal cancer
**MDT**	Multi-disciplinary team
**GP**	General Practitioner

Governance Statement:

This study will adhere to the conditions and principles which apply to all clinical studies as outlined in the EU Directive 2001/20/EC and Good Clinical Practice. It will be conducted in concordance with the protocol, the Data Protection Act 1998, sponsors' Standard Operating Procedures and other regulatory requirements as appropriate.

Vision Statement:

It is our goal to save the lives of women with Lynch syndrome (LS) by defining the burden of the disease in women newly diagnosed with endometrial cancer (EC), enabling their participation in colorectal screening programs and testing their first degree relatives for LS.

This is an exploratory study with the aim of contributing pilot data to

1. Define the prevalence of LS associated EC in a UK population of archived and prospectively collected of EC tumours
2. Identify the most effective method of screening women newly diagnosed with EC for LS
3. Develop a micro costing health economics model to provide primary intelligence into the costs of universal LS screening in women with EC

PETALS Study Schema

Prospective Study (n=200)

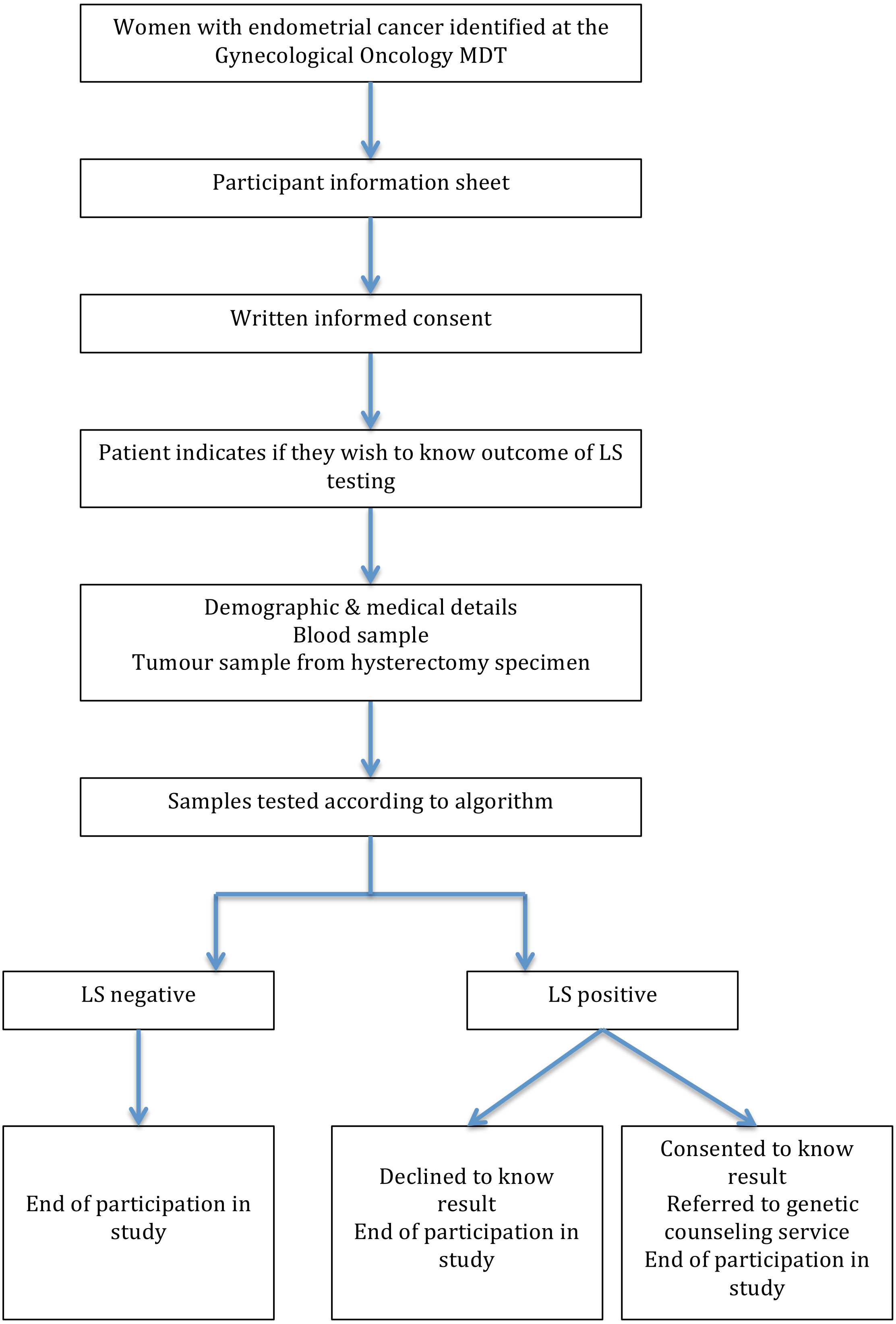

PETALS Study Schema

Retrospective Study (n=200)

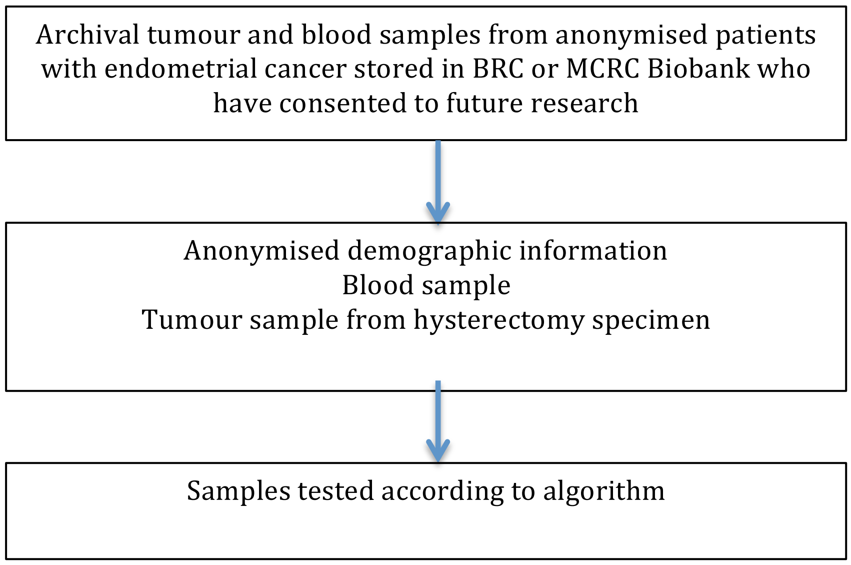

PETALS Testing Algorithm:

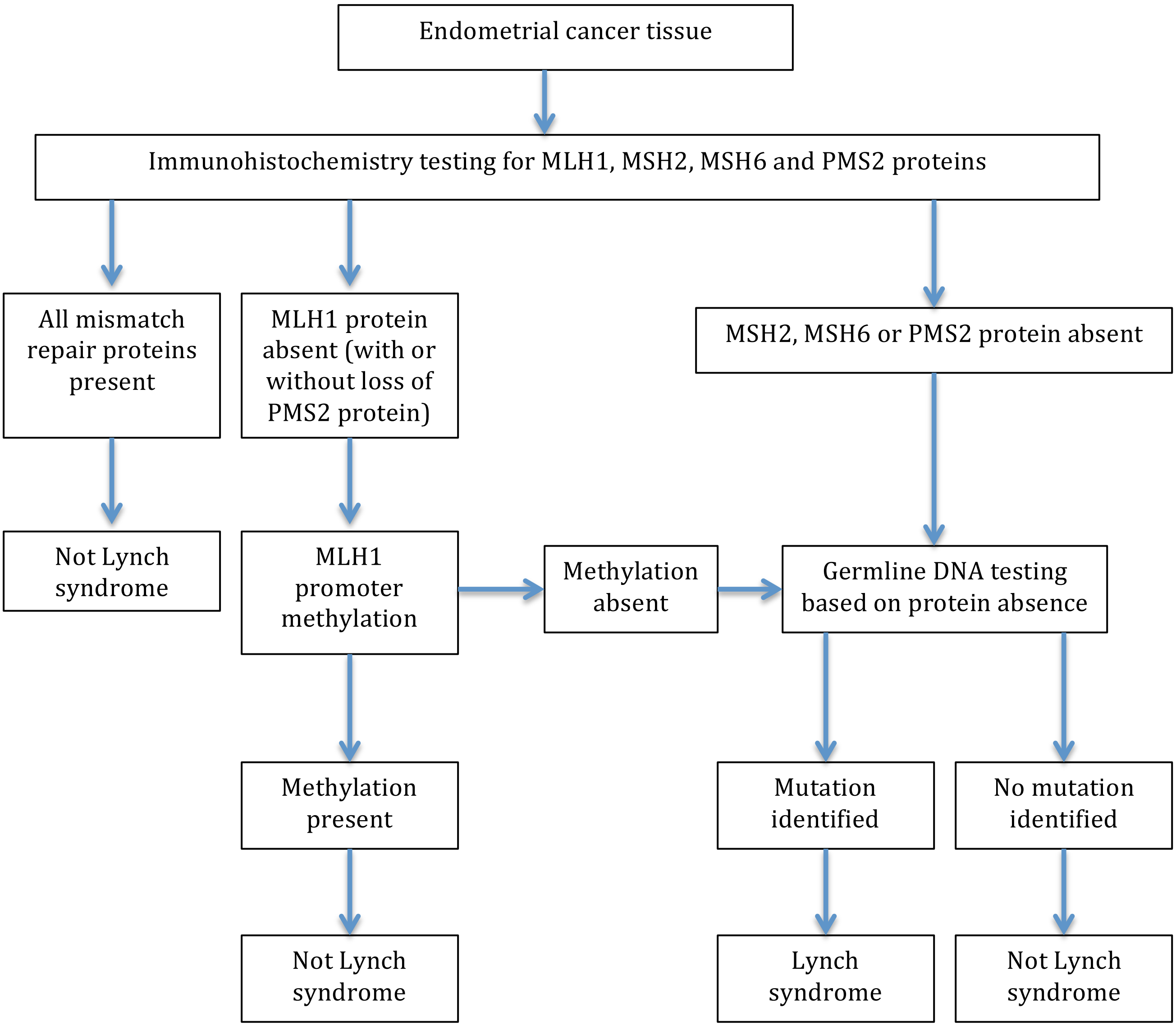

PETALS Study Synopsis

Study title:
Proportion of Endometrial Tumours Associated with Lynch Syndrome (PETALS)

Objective:
1. To determine the prevalence of LS in EC patients
2. To establish the most effective method of testing women with EC for LS
3. To measure costs of testing women with EC for LS
4. To determine differences in risk factor profile between LS-associated and sporadic EC patients

Number of participants:
- 200 anonymised patients for whom clinical data, tumour samples and blood DNA is already collected and stored for future research (retrospective study)
- 200 patients newly diagnosed with EC (prospective study)

Main inclusion criteria:
- Written informed consent
- Histological diagnosis of EC
- Clinical data, tumour and blood stored in Biobank for retrospective study

Primary outcome:
- Proportion of EC patients with LS [with a germline mutation in MLH1, MSH2, MSH6 or PMS2]

Secondary outcomes:
- Proportion of EC patients with tumour studies suggestive of LS (loss of MLH1, MSH2, MSH6 or PMS2 by immunohistochemistry and/or microsatellite instability (MSI) by PCR)
- Proportion of EC patients fulfilling Amsterdam II or revised Bethesda criteria for LS testing
- Costs of universal versus selected testing for LS in EC patients based on different triage strategies (Amsterdam II criteria, revised Bethesda criteria, positive tumour studies, germline mutation testing)
- Differences in risk factor profiles between LS-associated and sporadic EC (including age, BMI, reproductive, contraceptive and hereditary factors, histological subtype, stage and grade of tumour, biomarkers of poor prognosis)

Summary

Lay Summary:

Lynch syndrome is caused by specific gene faults that make cancer more likely to occur. Lynch syndrome runs in families. Bowel cancer and womb cancer are more common in people with Lynch syndrome. When we know someone has Lynch syndrome, we offer colonoscopy, a camera test to look inside the bowel, to remove any bowel polyps before they become cancerous. This has been shown to save lives.

As many as 1 in 10 women with womb cancer may have Lynch syndrome, although most do not know it. If they knew about the Lynch syndrome, they may choose to have regular screening by colonoscopy to remove bowel polyps. They may also help other family members find out whether they have Lynch syndrome. Womb cancer that develops in women with Lynch syndrome may behave differently and have a different outlook compared with womb cancer that occurs in women without Lynch syndrome. No one has looked at this before.

In this study, we will see how many patients with womb cancer have Lynch syndrome that they didn't know about. We can find this out by carrying out specific laboratory tests on the tumours. We will look at 200 women with womb cancer who gave clinical information about themselves, as well as blood and tumour samples for future research projects, when they came in for their hysterectomy. We will also test 200 women with new diagnoses of womb cancer as they come through the department for their surgery. We will carry out tests on the tumours and also blood tests to see if a gene fault is there. This will be done on the stored blood sample or, for current patients, genetic testing will be carried out through the genetic counseling service in our hospital. We will find out how many women with womb cancer have Lynch syndrome. We will also find out how straightforward it is to test women with womb cancer for Lynch syndrome. We will see whether patients are happy for their tumours to be tested for Lynch syndrome and whether they are prepared to go for genetic counseling. We will see whether they want to undergo colonoscopy to identify and treat bowel polyps. We will also see how much screening for Lynch syndrome in women with womb cancer is likely to cost. This will help us develop guidelines for which patients with womb cancer should be tested for Lynch syndrome in the future.

Abstract and summary of study design

This study will involve two groups of women with EC.

The first group will be **retrospective**, using stored anonymised clinical data, endometrial tissue and matched blood samples donated to the Biobank for future research. We will test 200 women with EC in the retrospective study.

The second group will be recruited **prospective**ly. Patients will be identified at the gynaecological oncology multi-disciplinary team (MDT) meeting at St Mary's Hospital. Those with a confirmed histological diagnosis of EC will invited to take part in the study when they attend for a routine appointment. Participation will involve providing detailed medical information about themselves, giving a blood sample and consenting to tumour studies on their endometrial tissue samples. Women will also be asked to complete a short questionnaire exploring the acceptability of testing for LS in EC. Women will choose whether they do or do not wish to know the results of any genetic tests that are undertaken. If they consent to know, they will be referred to the clinical genetics counseling service at St Mary's Hospital.

EC samples will be subjected to microsatellite instability index testing (MSI) by PCR and immunohistochemistry (IHC) testing to identify loss of MLH1, MSH2, MSH6 and PMS2 protein expression. If tumour studies suggest LS, reflex testing on the stored blood sample will be undertaken to establish the diagnosis of LS. This will be carried out anonymously for the retrospective patients and those prospective patients who did not wish to know the results.

<u>Study design</u>

Background

Lynch Syndrome (previously known as hereditary non-polyposis colorectal cancer, HNPCC) is an autosomal inherited cancer susceptibility syndrome. It was first described over a century ago[1]. The underlying pathogeneses of the disease is ineffective mismatch repair (MMR) of DNA, which leads to microsatellite instability and an increased risk of oncogenic mutations. The genes affected are MLH1, MSH6, MSH2 and PSM2[2]. . The products of these genes are crucial in stabilization and recruitment of the protein complexes responsible for DNA repair[3,4]. Other genes have been implicated, however these 'un-classical' loci remain controversial[5].

The estimated population incidence of LS is 1:660 - 1:2000[6]. LS individuals have a strong predisposition to cancers of the colon, uterus, ovary, stomach, pancreases, brain, hepatobiliary tract and ureoepithelial tract[1]. In addition LS patients can often present with multiple and synchronous primary malignancies[7]. LS patients typically present at an early age and have a strong family history of cancer; however, this not always the case. Various clinical criteria exist in order to identify potential LS individuals with the Amsterdam Criteria being the most widely cited. However, even in its revised form, sensitivity of the Amsterdam Criteria remains poor at 52%[8].

LS individuals represent 5% of all colorectal cancers (CRC)[9]. Accordingly, the vast majority of the literature on the condition explores its colonic manifestations. This is further compounded by the fact colon cancer is often the terminal event in the individual's life. The need to understand the role of LS in colorectal cancer has resulted in data that established the incidence of LS in CRC, a national screening program for LS amongst newly diagnosed CRC patients, and the identification of a unique tumour biomarker of LS associated CRC. Furthermore, those identified as having LS are offered a nationally agreed screening program to reduce their risk of dying from CRC. None of these benefits exist, however if their first malignancy is endometrial.

The sentinel cancer for LS females is commonly EC[10]. Women with LS have a lifetime risk of EC of around 50-60%[11]. In addition, the incidence of EC is rising with 8,475 new diagnoses in the UK last year[12]. It is now the most common gynaecological malignancy[13]. Currently there is no UK data on the incidence of LS within EC. Work outside of the UK has quoted the incidence rate of LS within the EC population of between 1-9% [5,6,14,15]. However these studies are underpowered, utilize varying inclusion criteria or are difficult to generalize to the UK due to their ethnic composition. Of note there remains no agreed criteria on which to screen EC for LS within in the UK[16]. These factors make planning and costing for a programme of screening EC for LS impractical. However, without such a programme, the diagnosis of LS will be missed leading to potentially avoidable deaths from synchronous or metachronous primary malignancies, for example of the colon.

Rational and objectives:

This study aims to provide pilot data to inform a future national screening programme for LS in women diagnosed with EC. It will provide the first UK prevalence data of LS-associated EC. It will determine the most effective method of testing for LS amongst women with EC (using tumour studies and/or germline mutation testing in all or certain groups of women with EC). It will provide costing data to inform a subsequent health economics modeling study to assess the cost effectiveness of such a screening programme. In addition it will explore the uptake rates of an offered screening programme and patient anxieties around screening. Through the collection of secondary outcome data we will also explore the impact of known risk factors for EC and the histological features of the tumour and its correlation with a LS diagnosis. In doing so we hope to address the inequality that exists between LS associated CRC and LS associated EC.

Retrospective Study Entry
Patients are eligible if all of the following inclusion criteria are met:

- Age >18 years
- Consent for future research on donated samples
- Clinical data, tumour and blood stored in Biobank

Prospective Study Entry
Patients are eligible if all of the following inclusion criteria are met:

- Age >18 years
- Able to give informed consent regarding sample donation and genetic testing
- Histological diagnosis of endometrial cancer

Participation in the Prospective Study

Women diagnosed with endometrial cancer will be identified at the gynaecology oncology multidisciplinary team (MDT) meeting. Women will be invited to take part when they attend their routine outpatient clinic appointment. They will be given a participant information sheet (PIS) and the opportunity to ask questions about the study. Written informed consent will be obtained.

Taking part in the study will involve providing detailed medical information about themselves, a blood sample and consent for tumour studies to be carried out on their tumour sample. They will be asked to complete a questionnaire exploring their motivations, concerns and understanding of the screening process. They will be asked whether they wish to know the outcome of the tumour and blood analyses. That is, if they have a positive or negative result would they like to know? Those who wish to know will be informed by letter, telephone call or in clinic (if tests are negative), according to patient preference, or invited for genetic counselling at the centre for Genomic Medicine based at St Mary's hospital (if tests are positive). Those who do not wish to know will receive no further information about their test results. Tumour and blood analyses will be conducted in an anonymised fashion to protect the identity of all participants.

A 10ml blood sample will be taken at recruitment. The specimen will be processed and stored for future analysis in the Biobank. The hysterectomy specimen will be processed according to standard protocols for the clinical care of the patient. Additional samples ('blocks') of the tissue will be taken, processed and stored in the pathology department or gynaecological oncology research laboratory until needed for analysis. Where hysterectomy

is not performed, the endometrial biopsy specimen used to diagnose EC will be used for the tumour analyses.

Assessments

- Detailed medical history (including age, BMI, reproductive, contraceptive and hereditary factors, histological subtype, stage and grade of tumour, biomarkers of poor prognosis)
- Tumour analyses (MMR protein loss and MSI testing by IHC and PCR respectively)
- Blood DNA testing (for mutations in MMR genes)

Additional assessments in PROSPECTIVE STUDY ONLY
- Preference to know result of tumour and blood analyses
- Questionnaire (Appendix 1)

Sample handling and storage:

Blood samples will be processed and stored in the BRC Biobank pending analysis. Samples will be stored anonymously, with no patient-identifying information on any of the sample tubes or vials.

Endometrial tumour samples will be stored in the Clinical Pathology Department or Gynaecological Oncology Research Laboratories (5th floor St Mary's) pending analysis. Both laboratories have a Human Tissue Authority License.

All tumour specimens will be formalin fixed and paraffin embedded (FFPE) and processed as per standard operating procedures. Tumour analyses will be performed in the Clinical Pathology Department or the Gynaecological Oncology Research Laboratories at St Mary's Hospital. Where hysterectomy is not performed, the diagnostic tumour blocks will be used. These may need to be transferred to St Mary's from other hospitals. Tumour blocks will be returned to the appropriate Pathology Department afterwards.

All data created from the analyses of these samples will be handled and stored according to the Data Protection Act 1998.

Tissue and blood analyses:

Immunohistochemistry (IHC) will be used to assess the presence/absence of the following mismatch repair (MMR) proteins:
- MLH1
- MSH2
- MSH6
- PMS2

Methylation studies will be conducted as per the testing algorithm on page 7 of this protocol. PCR will be used to measure microsatellite instability (MSI).

If the tumour analyses indicate possible Lynch syndrome (see testing algorithm, p7), the stored blood sample will undergo reflex genetic testing for a germline mutation in one or more of the MMR genes. These analyses will be conducted in the Clinical Genetics

Laboratory on the 6th floor of St Mary's Hospital. Patients in the prospective study who have consented to know the results of their tumour studies will be offered referral for genetic counseling and the outcome of their germline mutational analysis will be provided within the context of this clinical service.

In addition to those with positive tumour analyses, patients who meet the revised Bethesda or Amsterdam II criteria for LS testing (Appendix 2) will undergo germline mutational testing for LS. This will allow us to determine the accuracy of each approach for triaging women with EC for LS testing. Additional germline mutation testing will be conducted in a randomly selected cohort of the entire study population to determine the accuracy of the tumour studies, revised Bethesda and Amsterdam II criteria at selecting patients at 'high risk' of LS.

Potential for harm

Being diagnosed with cancer is a distressing and difficult time for most patients and their families. Patients will be given the opportunity to hear about our research and those who are eligible will be invited to take part. We will respect the right of patients to decline to participate and at no time will patients be pressurized.

Introducing the concept of a hereditary cause for some EC may cause distress and we will be sympathetic to this. Some patients may choose not to know the outcome of any tumour analyses or genetic testing that is done as part of this project. These wishes will be respected and all analyses will be conducted without patient identifiers in an anonymised way to protect all our participants' anonymity. Those who do wish to hear the outcome of their analyses will be informed in person, by telephone call or by letter, according to patient preference, if the result is negative for LS. Where the result is positive for LS, the patient will be offered referral to the Clinical Genetics Department for disclosure, support and advice regarding testing of family members and ongoing cancer screening and surveillance (eg for colorectal cancer).

LS is tested for in women with EC on an ad hoc and patchy basis currently and there are no agreed guidelines for who would benefit from testing and how this should be done. Our study will provide the first UK data to support routine testing for LS in some or all EC patients and will help improve the care of future patients with EC.

Unexpected findings

Patients found to have LS will be managed according to their expressed preference at the time of consent. If they wish to know a positive result they will be referred to the Clinical Genetics Department of the Manchester Centre of Genomic Medicine for further management. A negative result will be disclosed by letter, telephone call or in clinic, according to patient preference. No further action will be taken if the patient did not wish to know the outcome of their tests.

If during the course of clinical data collection, clinically important information is disclosed such as a high alcohol intake or a high depression score, the patient will be advised to contact their GP or, with their consent, managed as per local guidelines. We will offer to contact the GP in writing on their behalf about any non urgent important clinical issues. If an unexpected finding is discovered it will be discussed with the senior clinicians involved in the project leading to an individualized management schema.

Safety reporting

Adverse events

An adverse event is the appearance or worsening of any undesirable sign, symptom, or medical condition occurring after the study has commenced, even if not considered to be related to the procedures involved. Medical conditions/diseases present before starting the study will only be considered as adverse events if they worsen after the start of the study. Abnormal laboratory values or test results constitute adverse events only if they induce clinical signs or symptoms, are considered clinically significant, or require therapy.

The occurrence of adverse events will be sought by non-directive questioning of the patient during the study. Adverse events also may be detected when they are volunteered by the patient or through physical examination, laboratory test, or other assessment. As far as possible each adverse event will be evaluated to determine:

1. The severity (mild, moderate, severe)
2. Its relationship to the procedure performed
3. Its duration
4. Action taken (no action taken; medication taken; non-drug therapy given; hospitalisation required, surgery required)
5. Whether it is **serious**, where a serious adverse event (SAE) is defined as one which:
    - Is fatal or life-threatening
    - Results in persistent or significant disability/incapacity
    - Constitutes a congenital anomaly/birth defect
    - Requires prolonged hospitalisation (except where it is for routine treatment/monitoring, elective or pre-planned treatment not related to study, for social or respite reasons)
    - Is medically significant i.e. defined as an event that jeopardises the patient or may require medical or surgical intervention to prevent one of the above

Unlike routine safety assessments, SAEs are monitored continuously and have special reporting requirements (see below).

All adverse events will be recorded in detail and treated appropriately. Such treatment may include changes in study protocol including possible interruption or discontinuation, changes in the frequency or nature of assessments, hospitalisation, or any other medically required intervention. Once an adverse event is detected it will be followed until its resolution, and assessments will be made at each visit (or more frequently if necessary) of any changes in severity, the suspected relationship to the investigational procedure, the interventions required to treat it, and the outcome.

Evaluation of AEs and SAEs

Seriousness, causality, severity and expectedness will be evaluated for each AE. Cases that are considered serious, possibly, probably or definitely related to study interventions (i.e. serious adverse reactions, SARs) and unexpected (i.e. SUSARs) should be reported as described below.

Assessment of Seriousness

The Investigator should make an assessment of seriousness as defined above (see definitions).

Assessment of Causality

The Investigator must make an assessment of whether the AE/SAE is likely to be related to procedures according to the following definitions. All AEs/SAEs judged as having a reasonable suspected causal relationship (e.g. possibly, probably, definitely) to the study investigation will be considered as ARs/SARs..

Unrelated: an event is not considered to be related to the study procedure
Possibly: although a relationship to the study procedure cannot be completely ruled out, the nature of the event, the underlying disease, concomitant medication or temporal relationship makes other explanations possible.
Probably: the temporal relationship and absence of a more likely explanation suggest the event could be related to the study procedure.
Definitely: The known effects of the study procedure suggest that it is the most likely cause.

Alternative causes such as natural history of the underlying disease, other risk factors and the temporal relationship of the event to the procedure should be considered and investigated.

Assessment of Severity
The Investigator will make an assessment of severity for each AE/SAE and record this on the Adverse Event (AE) Form according to one of the following categories:
Mild: an event that is easily tolerated by the participant, causing minimal discomfort and not interfering with every day activities.
Moderate: an event that is sufficiently discomforting to interfere with normal everyday activities.
Severe: an event that prevents normal everyday activities.

Assessment of expectedness
If an event is judged to be an AR/SAR, the evaluation of expectedness will be made based on knowledge of the intervention and its known complications.

Serious Adverse Event (SAE) reporting
Any SAE will be reported by the study team (including a completed SAE form) within 24 hours of first knowledge to the Sponsor. The study team will ensure that the patient is appropriately treated. They will also determine whether the SAE is a SUSAR (Suspected Unexpected Serious Adverse Reaction). If it is deemed to be a SUSAR it will be reported immediately to the Sponsor. The Research Ethics Committee will be informed in accordance with Study regulations. An annual safety report will be sent by the study team to the Ethics Committee and Sponsor. Completed initial and follow-up Serious Adverse Event forms should be faxed to the sponsor on 0161 276 5766 and addressed 'For the attention of the Quality Manager'. Alternatively, scanned forms can be emailed to adverse.events@cmft.nhs.uk.

Regulatory Reporting Requirements
The sponsor, or their delegate, has a legal responsibility to notify the Research Ethics Committee that approved the study. Fatal or life threatening SUSARs will be reported no later than 7 calendar days, with a further 8 days for follow up information. All other SUSARs will be reported no later than 15 calendar days after the Sponsor is first aware of the reaction.

Follow up procedures
After initially recording an AE or recording and reporting an SAE, the study team is required to follow each participant until resolution. Follow up information on an SAE should be

reported to the Sponsor. AEs still present in participants at the last study visit should be monitored until resolution of the event or until no longer medically indicated.

Criteria for premature termination of study

These criteria include new safety data, or concerns from safety data (number and nature of SUSARs); or evidence from other studies.

Flowchart for SAE assessments

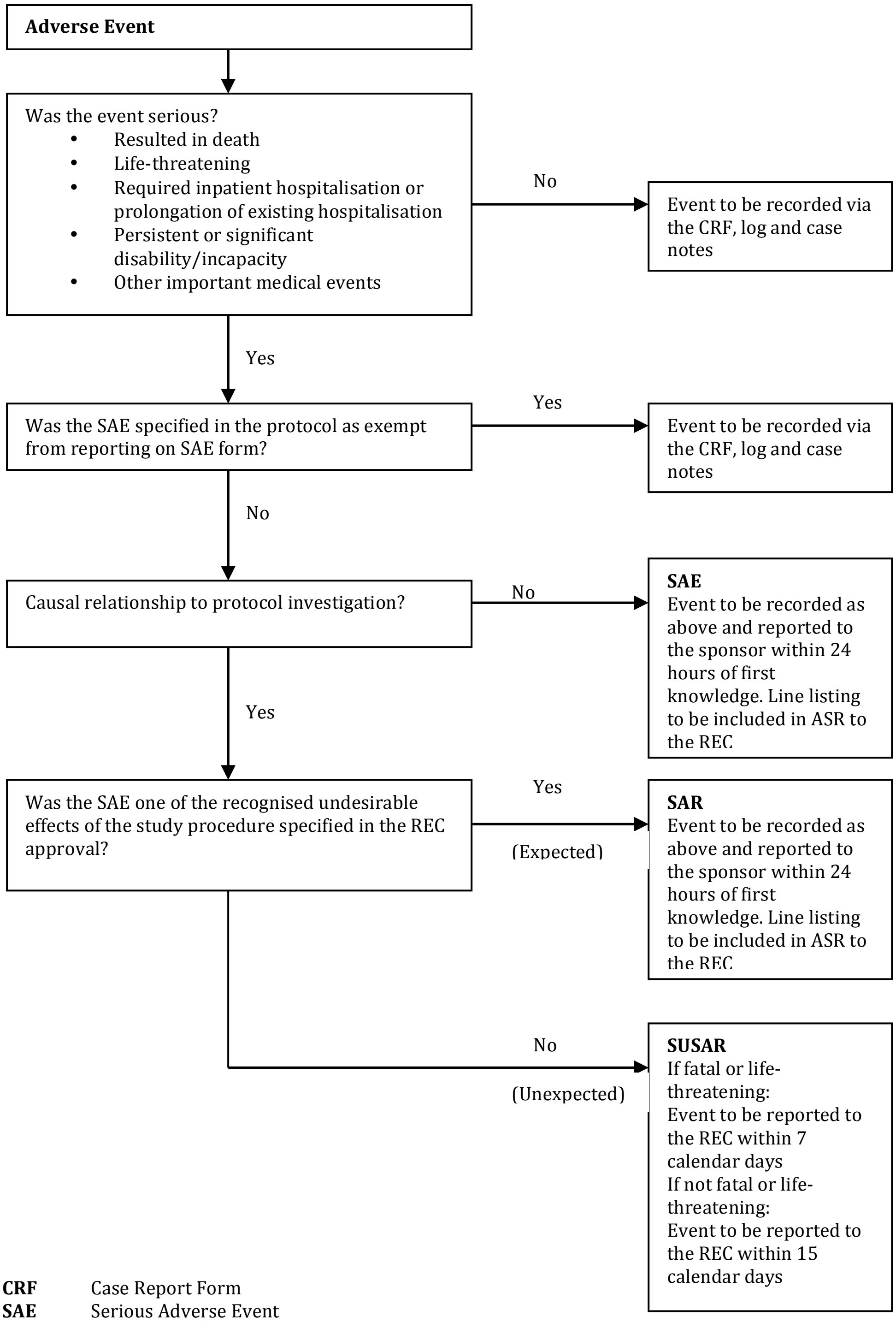

**CRF**	Case Report Form
**SAE**	Serious Adverse Event
**SAR**	Serious Adverse Reaction
**SUSAR**	Suspected Unexpected Serious Adverse Reaction
**REC**	Research Ethics Committee

Study Conduct and Monitoring

Data Handling
All data will be handled and stored according to the Data Protection Act 1998. CRFs and a study log will be kept in a locked filing cabinet in a locked research office on the 5th floor of St Mary's. Only members of the research team have access to this room. CRFs will be labelled with study specific IDs and no patient-identifying information will be stored in these files. Data will also be stored on a University networked PC in the same office. The data will be stored in a password-restricted fashion that is only accessible to members of the research team.

Loss to follow up
If a participant is lost to follow up, the GP will be contacted to obtain information on the participant's status.

Participant withdrawal
In consenting to the study, participants are consenting to study procedures, follow-up and data collection. They may withdraw from the study whenever they wish. Withdrawal from the study may also be necessary if the responsible physician deems it to be in the best interest of the patient. If a participant explicitly withdraws consent to have any data recorded, their decision must be respected and recorded on the withdrawal form. Details of the withdrawal form should be noted in the participant's records.

Study end point
The study will end when all 400 samples have been analysed as per the testing algorithm.

Informed consent, ethical and regulatory considerations
The protocol will have the favourable opinion of a Research Ethics Committee (REC) as part of the Clinical Study Authorisation. All participants will be informed of the aims of the study, the known possible adverse events, the procedures and possible hazards to which they may be exposed. They will be informed of the strict confidentiality of their data, but that their medical records may be reviewed for study purposes by authorised individuals other than their treating physician.

The participant's consent to take part in the study will be obtained after a full explanation has been given of the procedures involved and the implications of these. Participants will be given sufficient time after being given the study Participant Information Sheets (PIS), to consider and discuss participation in the study with friends and family. A contact number will be given to the participant should they wish to discuss any aspect of the study. Following this, the recruiting investigator will determine that the participant is fully informed of the study and their participation is in accordance with UK regulations. Participants will always be asked to sign and date a consent form. One copy will be given to the participant but the original copy will be kept in the study site file and a further copy will be kept with participant's hospital notes.

The right of the participant to refuse to take part in the study without giving reasons must be respected. After the participant has entered the study, the investigator must remain free to give alternative treatment to that specified in the protocol, at any stage, if he/she feels it to be in the best interest of the participant. However, the reason for doing so will be recorded and the participant will remain within the study for the purpose of follow up and data analysis. Similarly, the participant must remain free to withdraw at any time from the protocol procedures without giving reasons and without prejudicing her further treatment.

The study team will:

- Have in place arrangements to adhere to the principles of Good Clinical Practice (GCP)
- Keep a copy of all essential documents (as defined by GCP) and ensure appropriate archiving and destruction once the study has ended
- Take appropriate urgent safety measures
- Observe reporting requirements to the Ethics Committee as required.

Sponsorship and indemnity

The sponsor of the study is Central Manchester University Hospitals NHS Foundation Trust. CMFT will be responsible for ensuring that the clinical study is performed in accordance with the following:

- Declaration of Helsinki (South Africa 1996)
- Good Clinical Practice
- Research Governance Framework for Health and Social Care 2001 and subsequent amendments

The Sponsor will ensure the following:
1. That appropriate ethics committee opinion has been sought
2. R&D approval at all sites has been obtained prior to the start of recruitment
3. Amendments have been discussed and reviewed prior to submission for approval
4. That the REC is informed when the study has ended
5. That Annual Safety Reports are submitted to REC within specified timeframes
6. Annual Progress Reports are submitted to the REC within specified timeframes
7. That urgent safety measures are taken as appropriate
8. A report is submitted within 12 months of the end of study notification to the REC

10.1 Negligent harm

CMFT continues to have a duty of care to its patients, whether or not the patient is involved in a clinical study. The Sponsor shall indemnify the Site against claims arising from the negligent acts and/or omissions of the Sponsor or its employees in connection with the Clinical Study (including the design of the Protocol to the extent that the Protocol was designed solely by the Sponsor and the Site has adhered to the approved version of the Protocol) save to the extent that any such claim is the result of negligence on the part of the Site or its employees.

Data protection

We will act to preserve participant confidentiality and will not disclose or reproduce any information by which participants could be identified. Data will be stored in a secure manner and our studies are registered in accordance with the Data Protection Act 1998.

Publication policy

Data will be analysed and published as soon as possible. All contributing investigators will be included in any such publication and the draft manuscript will be approved by the sponsor and all authors prior to submission.

Finance

We have funding from the Medical Research Council (MRC) to undertake this study.

References:

1. Douglas, J. A. *et al.* History and molecular genetics of Lynch syndrome in family G: a

century later. *JAMA* **294,** 2195–2202 (2005).
2. Lynch, H. T. *et al.* History of the International Collaborative Group on Hereditary Non Polyposis Colorectal Cancer. *Fam. Cancer* **2,** 3–5 (2003).
3. Peltomäki, P. Epigenetic mechanisms in the pathogenesis of Lynch syndrome. *Clin. Genet.* **85,** 403–412 (2014).
4. Fishel, R. & Kolodner, R. D. Identification of mismatch repair genes and their role in the development of cancer. *Curr. Opin. Genet. Dev.* **5,** 382–395 (1995).
5. Walsh, M. D. *et al.* Molecular, pathologic, and clinical features of early-onset endometrial cancer: identifying presumptive Lynch syndrome patients. *Clin. Cancer Res.* **14,** 1692–1700 (2008).
6. la Chapelle, de, A. The incidence of Lynch syndrome. *Fam. Cancer* **4,** 233–237 (2005).
7. Vasen, H. F. A. *et al.* Guidelines for the clinical management of Lynch syndrome (hereditary non-polyposis cancer). *J. Med. Genet.* **44,** 353–362 (2007).
8. Park, J.-G. *et al.* Suspected HNPCC and Amsterdam criteria II: evaluation of mutation detection rate, an international collaborative study. *Int J Colorectal Dis* **17,** 109–114 (2002).
9. Garre, P. *et al.* Cancer risk and overall survival in mismatch repair proficient hereditary non-polyposis colorectal cancer, Lynch syndrome and sporadic colorectal cancer. *Fam. Cancer* **13,** 109–119 (2014).
10. Wang, Y. *et al.* Lynch syndrome related endometrial cancer: clinical significance beyond the endometrium. *J Hematol Oncol* **6,** 22 (2013).
11. Dunlop, M. G. *et al.* Cancer risk associated with germline DNA mismatch repair gene mutations. *Hum. Mol. Genet.* **6,** 105–110 (1997).
12. Crosbie, E. & Morrison, J. The emerging epidemic of endometrial cancer: Time to take action. *Cochrane Database Syst Rev* **12,** ED000095 (2014).
13. Evans, T. *et al.* Differential trends in the rising incidence of endometrial cancer by type: data from a UK population-based registry from 1994 to 2006. *British Journal of Cancer* **104,** 1505–1510 (2011).
14. Hampel, H. *et al.* Screening for Lynch syndrome (hereditary nonpolyposis colorectal cancer) among endometrial cancer patients. *Cancer Res.* **66,** 7810–7817 (2006).
15. Egoavil, C. *et al.* Prevalence of Lynch syndrome among patients with newly diagnosed endometrial cancers. *PLoS ONE* **8,** e79737 (2013).
16. RCOG. Management of Women with a Genetic Predisposition to Gynaecological Cancers. 1–9 (2015).
17. Shia, J., Black, D., Hummer, A. J., Boyd, J. & Soslow, R. A. Routinely assessed morphological features correlate with microsatellite instability status in endometrial cancer. *Hum. Pathol.* **39,** 116–125 (2008).
18. Vadaparampil, S. T., Ropka, M. & Stefanek, M. E. Measurement of psychological factors associated with genetic testing for hereditary breast, ovarian and colon cancers. *Fam. Cancer* **4,** 195–206 (2005).
19. Boland, C. R. *et al.* A National Cancer Institute Workshop on Microsatellite Instability for cancer detection and familial predisposition: development of international criteria for the determination of microsatellite instability in colorectal cancer. in *Cancer Res.* **58,** 5248–5257 (American Association for Cancer Research, 1998).
