# Supplementary material for: The proportion of endometrial tumours associated with Lynch syndrome (PETALS): A prospective cross-sectional study
Source: PLoS Med. 2020 Sep 17;17(9):e1003263. doi: 10.1371/journal.pmed.1003263 (PMC7497985; doi:10.1371/journal.pmed.1003263)
Supplement: S1 Text — (DOCX) [file pmed.1003263.s004.docx]

**The Proportion of Endometrial Tumours Associated with Lynch Syndrome:**

**a prospective diagnostic test accuracy study of unselected screening of endometrial cancer for Lynch syndrome (PETALS study)**

**Supporting Information**

Table of Contents

Appendix 1 2

Methods 2

Extended methods for immunohistochemistry 2

Examples of MMR immunohistochemical staining 3

Figure 1 Examples of MMR loss on IHC (Complete and Patchy) 3

Extended methods for germline analysis 3

Primers used for germline analysis 4

Table 1 *MSH2* primer sequences for NGS – chromosome 2 5

Table 2 *MSH6* primer sequences for NGS – chromosome 2 6

Table 3 *MLH1* primer sequences for NGS – chromosome 3 7

Somatic genomic analysis methods 8

# **Appendix 1**

## **Methods**

### Extended methods for immunohistochemistry

MLH-1 Protocol Details

Antibody: Anti-MLH-1 (M1) Mouse Monoclonal

Manufacturer: Ventana

System: Automated Ventana Benchmark ULTRA

Detection Kit: OptiView DAB IHC Detection System

Antigen Retrieval: 24 minute heat in CC1 (Tris based pH8.4 buffer)

Antibody concentration: RTU (Ready to use)

Antibody Incubation: 16 minute at room temperature.

Counterstain: Haematoxylin 12 min, Bluing Reagent 4 min.

Positive Tissue Control: Colon

MSH2 Protocol Details

Antibody: MSH2 (G219-1129) Mouse Monoclonal

Manufacturer: Cell Marque

System: Automated Ventana Benchmark ULTRA

Detection Kit: OptiView DAB IHC Detection System

Antigen Retrieval: 32 minute heat in CC1 (Tris based pH8.4 buffer)

Antibody concentration: 1+50

Antibody Incubation: 16 minute at room temperature.

Counterstain: Haematoxylin 12 min, Bluing Reagent 4 min.

Positive Tissue Control: Colon

MSH6 Protocol Details

Antibody: MSH6 (SP93) Rabbit Monoclonal

Manufacturer: Cell Marque

System: Automated Ventana Benchmark ULTRA

Detection Kit: OptiView DAB IHC Detection System

Antigen Retrieval: 32 minute heat in CC1 (Tris based pH8.4 buffer)

Antibody concentration: 1+50

Antibody Incubation: 16 minute at room temperature.

Counterstain: Haematoxylin 12 min, Bluing Reagent 4 min.

Positive Tissue Control: Colon

PMS2 Protocol Details

Antibody:                               Anti-PMS2

Manufacturer:                          Ventana (760-4531)

System:                                   Automated Ventana Benchmark ULTRA

Detection Kit:                         OptiView DAB IHC Detection System

Antigen Retrieval:                 64 minute heat in CC1 (Tris based pH8.4 buffer)

Antibody concentration:       RTU (Ready to use)

Antibody Incubation:               32 minute at room temperature

Additional Kit: Optiview Amplification 4 min, Optiview Amplification Multimer 4 Min

Counterstain:                         Haematoxylin II 12 min, Bluing Reagent 4 min.

Positive Tissue Control:         Colon

### Examples of MMR immunohistochemical staining

F

D

B


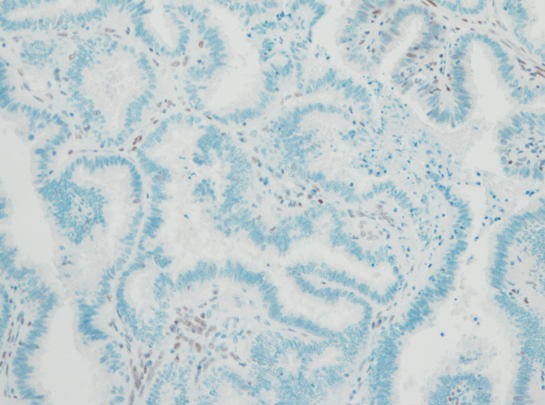

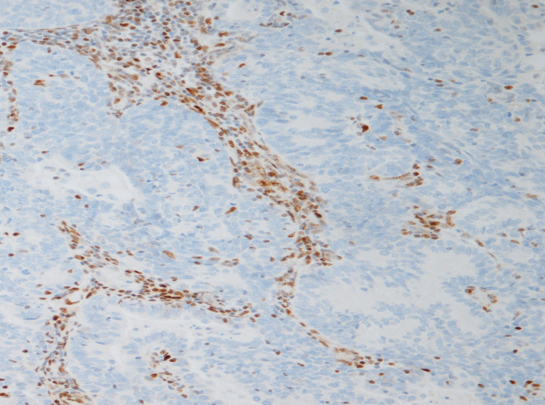

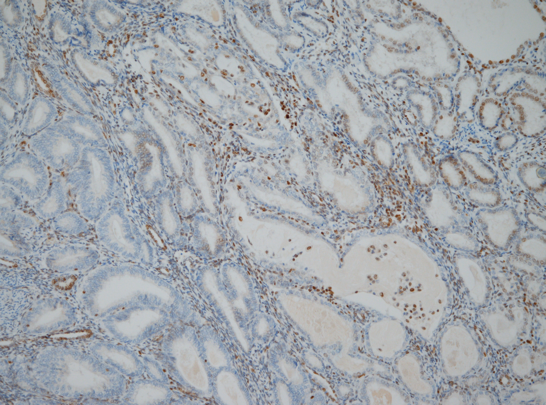

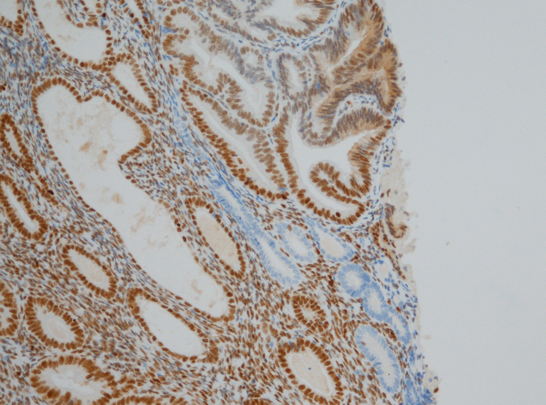


C

E

D

F


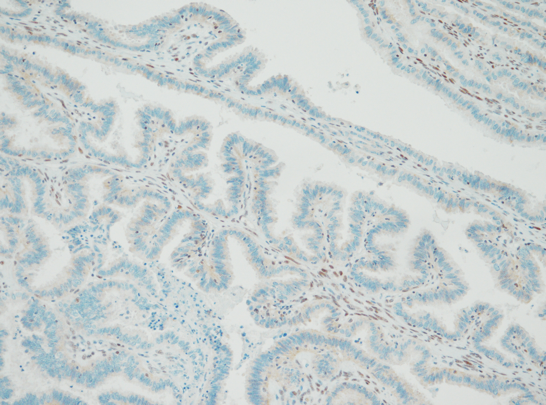

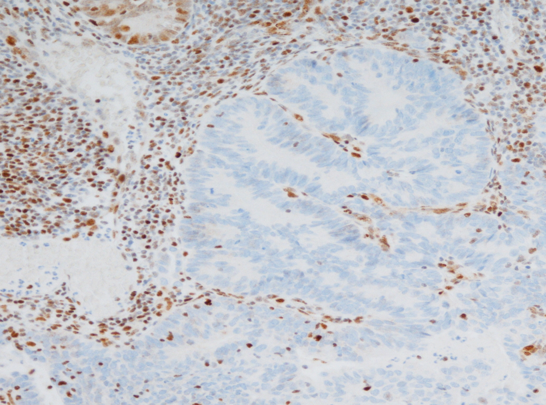


A

B

### Figure 1 Examples of MMR loss on IHC (Complete and Patchy)

A: Complete MLH1 loss; B : Complete MHS2 loss; C: Complete MSH6 loss; D: Complete PMS2 loss; E and F: Examples of patchy MMR IHC loss.

### Extended methods for germline analysis

Blood samples were taken after patient counselling and consent. These were stored in Ethylenediaminetetraacetic acid (EDTA) tubes. DNA extraction was performed on the Chemagen Chemagic 360 (Perkin Elmer) with a liquid handling integration as per manufactures specifications. Post extraction concentration testing performed via Qubit Fluorometric Quantification (ThermoFisher Scientific).

.

Germline screening of *MLH1*, *MSH2* and *MSH6* was performed by next generation sequencing, using a long range PCR enrichment approach across 22 amplicons varying in size from 1.3 kb to 6.7 kb, Nextera XT library preparation and sequencing on a MiSeq using Illumina MiSeq v2 2 x 150 bp paired end reads. Copy number analysis was assessed using current version of MRC-Holland MLPA probe kits P003 for *MLH1* and *MSH2* and P072 for *MSH6*. *MSH6* exon 1 was analysed by Sanger sequencing due to poor coverage of this region using NGS technology.

Bioinformatic analysis used an in house developed and validated bioinformatics analysis pipeline validated to detect low level mosaic calls down to 4% variant allele fraction and used a software consensus between VarScan v2.3.6 and DREEP v0.7. Large indel events were assessed using Pindel (v0.2.4.t). In addition, an assessment was made of the frequency of each variant called within each batch of samples analysed.  This aids in the interpretation of whether a variant is real or an artefact.

Variants identified bioinformatically were assessed for clinical relevance following ACMG/ACGS guidelines on variant interpretation. Pathogenic, likely pathogenic and variants of unknown clinical significance identified by next generation sequencing were all confirmed by Sanger sequencing analysis.

*PMS2* analysis was conducted by an external clinical laboratory with the use of MRC-Holland MLPA probe kits P003. More details regarding their methods can be found at <http://www.leedsth.nhs.uk/a-z-of-services/the-leeds-genetics-laboratory/>.

### Primers used for germline analysis

The primers designed for this assay are shown in Tables 1-3 below. These were designed using the following reference sequences for *MSH2, MSH6*, and *MLH1* genes:

- ***MSH2* Ensembl transcript**  ENSG00000095002. NM_000251.2
- ***MSH6* Ensembl transcript** ENSG00000116062. NM_000179.2
- ***MLH1* Ensembl transcript** ENSG00000076242. NM_000249.3

### Table 1 *MSH2* primer sequences for NGS – chromosome 2

|  | **Exon coverage** | **Primer name** | **Length (bp)** | **Amplicon size (bp)** | **Sequence** |
| --- | --- | --- | --- | --- | --- |
| *MSH2* | Exon 1 | *MSH2*_Ex1_F | 30 | 2779 | CACGTTTTAACAAAATACTGGGAGGAGGAG |
|  |  | *MSH2*_Ex1_R | 30 |  | AACCACTGCACTTGGTCTGTCCTTTCTTAT |
| *MSH2* | Exon 2,3 | *MSH2*_Ex2-3_F | 30 | 6070 | AAACACTGGAAGTTTTTGGGTTAGCATTGT |
|  |  | *MSH2*_Ex2-3_R | 30 |  | GCTAAAAATGCCCATCACATGTGTTATCTC |
| *MSH2* | Exon 4, 5, 6 | *MSH2*_Ex4-6_F | 30 | 4912 | GATAACACATGTGATGGGCATTTTTAGCAT |
|  |  | *MSH2*_Ex4-6_R | 33 |  | GTAATTTACCCACGATTACACACAATATGAACA |
| *MSH2* | Exon 7 | *MSH2*_Ex7_F | 30 | 2529 | AGCTGAGATTTTATTGACTTTGGGAAGCAG |
|  |  | *MSH2*_Ex7_R | 30 |  | TCAACTGTTAGAGACCACCAAGACCCTCTA |
| *MSH2* | Exon 8 | *MSH2*_Ex8_F | 30 | 3383 | ATTCATAAACCAGTGCCACCTCTGAATTTT |
|  |  | *MSH2*_Ex8_R | 30 |  | TTTTTAAAAGGGTGGGGGATTACTCCTGTA |
| *MSH2* | Exon 9, 10 | *MSH2*_Ex9-10_F | 30 | 5749 | CTAAGGTAGATCCTTGGTTTGGGCAACATA |
|  |  | *MSH2*_Ex9-10_R | 30 |  | AGGAGGATTCTAGTGTGCTGGAAAAACAAC |
| *MSH2* | Exon 11 | *MSH2*_Ex11_F | 30 | 2837 | ACTACAGGTGTTGCCCACTGCTTTGTATTT |
|  |  | *MSH2*_Ex11_R | 30 |  | GTTGCCCTGTCTCTTTAAGACCTGTTGATT |
| *MSH2* | Exon 12, 13 14 | *MSH2*_Ex12-14_F | 30 | 5260 | GAATTCTTCAAAAGTGTACTGAGGCCAGGT |
|  |  | *MSH2*_Ex12-14_R | 30 |  | TACATGTTCCTACCCCCAGACTGTGAATTA |
| *MSH2* | Exon 15, 16 | *MSH2*_Ex15-16_F | 30 | 5222 | GGTTGCACATGAGTGAGATAATCTTGGTTC |
|  |  | *MSH2*_Ex15-16_R | 30 |  | CACTTGGATATACGTTGGAGTGGAATTGTC |

### Table 2 *MSH6* primer sequences for NGS – chromosome 2

| **Gene** | **Exon coverage** | **Primer name** | **Length (bp)** | **Amplicon size (bp)** | **Sequence** |
| --- | --- | --- | --- | --- | --- |
| *MSH6* | Exon 2 | *MSH6*_Ex2_F | 30 | 3024 | AGGATGCACATTTATCCTGTAAACAAATGG |
|  |  | *MSH6*_Ex2_R | 30 |  | ACTTCCCAATTCTCTTTCCACCTGTATGTC |
| *MSH6* | Exon 3, 4 | *MSH6*_Ex3-4_F | 30 | 6701 | GCCATTGTGCCCAGCTAGTAAGTTTTTAAG |
|  |  | *MSH6*_Ex3-4_R | 30 |  | AAATCCTAGCTACCTAAGAGGCTGACACGA |
| *MSH6* | Exon 5, 6, 7, 8, 9, 10 | *MSH6*_Ex5-10_F | 30 | 5145 | TTGTTCTTTAAATGTCACGGCCAACATTAC |
|  |  | *MSH6*_Ex5-10_R | 30 |  | GCATTCAAAATCACCCCTAGAAAAAGTGAG |

### Table 3 *MLH1* primer sequences for NGS – chromosome 3

| **Gene** | **Exon coverage** | **Primer name** | **Length (bp)** | **Amplicon size (bp)** | **Sequence** |
| --- | --- | --- | --- | --- | --- |
| *MLH1* | Exon 1,2 | *MLH1*_Ex1-2_F | 29 | 4664 | AAAACTAGAGCCTCGTCGACTTCCATCTT |
|  |  | *MLH1*_Ex1-2_R | 30 |  | CTTAAATTGAACATGCCCCAAAATGAACTT |
| *MLH1* | Exon 3,4 | *MLH1*_Ex3-4_F | 30 | 6412 | CAACAAAAAGCCCTGAGACTGAATGAGATA |
|  |  | *MLH1*_Ex3-4_R | 30 |  | CCAGCAAATGTTGCTATTTACTTGCTGAGT |
| *MLH1* | Exon 5,6 | *MLH1*_Ex5-6_4F | 32 | 2709 | TCCAATTCAAATGATTATGGAAGTAGTGGAGA |
|  |  | *MLH1*_Ex5-6_4R | 30 |  | CCATCTGTACCAGCAGCGTAAGTCTATCAT |
| *MLH1* | Exon 7,8 | *MLH1*_Ex7-8_3F | 30 | 2553 | TCTAGTTGGCTGTTTTGATTCTAGGTGTGG |
|  |  | *MLH1*_Ex7-8_3R | 3 |  | CCATACACACTGAACCAGCAAACTCTTTTT |
| *MLH1* | Exon 9 | *MLH1*_Ex9_F | 30 | 3673 | ATTTCCATCATAGATTCCTAGCGGTCTCAG |
|  |  | *MLH1*_Ex9_R | 30 |  | TTTTCCAGAATTTCAGGCAGTACAAGTGTC |
| *MLH1* | Exon 10,11 | *MLH1*_Ex10-11_F | 30 | 4646 | GGCTCACTATTTAGCACACACACACGTCTA |
|  |  | *MLH1*_Ex10-11_R | 30 |  | TGTGTATCATAGTGCTCCAAGAGCCTTACC |
| *MLH1* | Exon 12 | *MLH1*_Ex12_F | 30 | 1357 | TTATACTTCAAATTTCGGGCAGAATTGCTT |
|  |  | *MLH1*_Ex12_R | 30 |  | ACCACTAGCTCACGTACTTCAGGGATTTTT |
| *MLH1* | Exon 13 | *MLH1*_Ex13_F | 30 | 1814 | CAAATATTTTCAAGCCCAAAATCAAGTGGT |
|  |  | *MLH1*_Ex13_R | 30 |  | CTGAGGCATAGAAAGGTCAGGAGTCTAAGG |
| *MLH1* | Exon 14, 15 | *MLH1*_Ex14-15_F | 30 | 4160 | TTTGCATTTGATTCTACTGGAGGAACAGTC |
|  |  | *MLH1*_Ex14-15_R | 30 |  | AGCCTAGGAGTTCAAAGCTTCAGTGACCTA |
| *MLH1* | Exon 16, 17, 18, 19 | *MLH1*_Ex16-19_F | 30 | 5404 | GCCTGTTCTGTTATTGAGAAAGGGGTATTG |
|  |  | *MLH1*_Ex16-19_R | 30 |  | TTCTAGAACTCCATCCCAGCTAAAACCAAC |

### Somatic genomic analysis methods

Four x 5uM thick FFPE sections or tumour cores were processed to extract genomic DNA using the Roche cobas® DNA Sample Preparation Kit. Double stranded DNA was quantitated using a Qubit fluorometer and samples normalised to 5 ng/μL concentration.

Library enrichment used a short amplicon approach using a 13 gene colorectal cancer custom design using a Qiagen GeneRead DNAseq Custom Panel v2. The panel includes 13 genes, *APC, BMPR1A, MSH6, SMAD4, MLH1, MSH2, MUTYH, POLD1, POLE,* *PTEN*, *STK11* *CDH1* and *CTNNB1* and loss-of-heterozygosity (LOH) analysis across the three mismatch repair genes (*MLH1*, *MSH2*, *MSH6*).

Library enrichment comprised 250 primer pairs split between 8 multiplex primer pools. 20ng of normalised FFPE derived DNA was amplified in each multiplex primer pool. For the colorectal cancer custom panel, the percentage of coding regions and immediate intron exon boundaries covered by the enrichment of key genes were; *MLH1* 100%, *MSH2* 99.99%, *MSH6* 99.99%, *APC* 99.99%, *CDH1* 99.98%

Following PCR based target enrichment, library preparation and purification followed a custom protocol using AMPure XP beads from Agencourt for size selection and Illumina TruSeq PCR Free indexes and reagents for indexing. The DNA library was then paired end sequenced on an Illumina MiSeq with v2 chemistry (2x150cycles).

Bioinformatic analysis used an in house developed bioinformatics analysis pipeline which was validated to detect low level mosaic calls down to 4% allele fraction and used a software consensus between VarScan v2.3.6 and DREEP v0.7. Large indel events were assessed using Pindel (v0.2.4.t). In addition, an assessment was made of the frequency of each variant called within each batch of samples analysed. This aids in the interpretation of whether a variant is real or an artefact.

Variants identified bioinformatically were assessed for clinical relevance following ACMG/ACGS guidelines on variant interpretation.
