# Supplementary material for: The proportion of endometrial tumours associated with Lynch syndrome (PETALS): A prospective cross-sectional study
Source: PLoS Med. 2020 Sep 17;17(9):e1003263. doi: 10.1371/journal.pmed.1003263 (PMC7497985; doi:10.1371/journal.pmed.1003263)
Supplement: S2 Text — MMR, mismatch repair. (DOCX) [file pmed.1003263.s005.docx]

**The Proportion of Endometrial Tumours Associated with Lynch Syndrome:**

**a prospective diagnostic test accuracy study of unselected screening of endometrial cancer for Lynch syndrome (PETALS study)**

**Supporting Information**

Table of Contents

Appendix 2 2

Reasoning for mutational analysis and mutation classification in unclear or unknown MMR mutations 2

Table 4 Indications for both germline and somatic analysis 2

Table 5 Reasoning for mutational classification. 3

# **Appendix 2**

## **Reasoning for mutational analysis and mutation classification in unclear or unknown MMR mutations**

###

### Table 4 Indications for both germline and somatic analysis

| **Germline tested cohort** | |  |  |
| --- | --- | --- | --- |
| **Characteristics** | |  |  |
| Age | 58 (SD 14.32) |  |  |
| BMI | 31 (IQR 25-38) |  |  |
| Endometrioid | 116 (85%) |  |  |
| **Indication** | **Number** | **No. germline *path_MMR*** | **No. germline *vus_MMR*** |
| Age | 35 | 0 | 4 |
| FHx | 12 | 0 | 0 |
| MMRd only | 16 | 7 | 3 |
| MSI-H only | 7 | 0 | 0 |
| MMRd and MSI-H | 18 | 9 | 3 |
| *MLH1*- hypermethylation | 26 | 0 | 0 |
| Patchy MMRd | 22 | 0 | 1 |
| Total | 136 | 16 | 11 |
| **Indication** | **Number** | **No. Somatic *path_MMR*** | **No. Somatic *vus_MMR*** |
| Age | 0 | NA | NA |
| FHx | 0 | NA | NA |
| MMRd only* | 5 | 4 | 0 |
| MSI-H only^ | 6 | 1 | 0 |
| MMRd and MSI-H^^ | 8 | 7 | 0 |
| *MLH1*-hypermethylation | 0 | NA | NA |
| Patchy MMRd** | 15 | 4 | 0 |
| Total | 34 | 16 | 0 |

| *Two complete MMRd only loss in non-germline path_MMR failed |
| --- |
| ^One MSI-H only sample had no material |
| ** Three samples in the patchy IHC samples failed. |
| ^^ One MMRd and MSI-H sample failed |

Abbreviations: BMI: body mass index, FHx: family history, IHC: immunohistochemistry, MSI-H: microsatellite instability MMRd- Mismatch repair deficient on immunohistochemistry

### Table 5 Reasoning for mutational classification.

| **PET ID** | **Mutation** | **Type** | **InSiGHT Class (Presumptive classifications in brackets)** | **Reason/s** | **Evidence** |
| --- | --- | --- | --- | --- | --- |
| 209 | *MSH6* c.3600A>G p.(lle1200Met) | MS | [3] | FH suggestive of LS, but prior as MS is not quite 0.5, so a large amount of other evidence would be needed to achieve a posterior >0.95. No tumour data supporting pathogenicity. |  |
| 101 | *MSH2* c.2120G>A p.(Cys707Tyr) | MS | [3/2] | Prior as MS is >0.5, supporting pathogenicity, but non-concordant IHC and frequency in a defined population of 0.33% (although <1%) does not support this. Mosaicism implies de novo, but paternity unconfirmed and MMRd inconsistent. | Somatic analysis of the tumour found no other pathogenic MMR mutations supporting a classification of 3 |
| 73 | *MSH2* c.1760-7delT | SS | [3] | Not previously reported as pathogenic. No tumour data supportive and FH inconsistent with LS. (Patient has very high BMI: likely cause of EC.) |  |
| 128 | *MSH6* c.3313G>T p.(Gly1105Ter) | NS | **[5]** | Coding sequence variation resulting in a stop codon i.e. a nonsense or frameshift alteration that is not after codon 743 in *MLH1* or after codon 888 in *MSH2*, and not in the last exon of *MSH6* or *PMS2*. |  |
| 215 | *MSH6* c.3004_3005delGG p.(Gly1002LeufsTer2) | FS | **[5]** | Coding sequence variation resulting in a stop codon i.e. a nonsense or frameshift alteration that is not after codon 743 in *MLH1* or after codon 888 in *MSH2*, and not in the last exon of *MSH6* or *PMS2*. |  |
| 16 | *MLH1* c.473delA p.(Asn158ThrfsTer2) | FS | **[5]** | Coding sequence variation resulting in a stop codon i.e. a nonsense or frameshift alteration that is not after codon 743 in *MLH1* or after codon 888 in *MSH2*, and not in the last exon of *MSH6* or *PMS2*. |  |
| 882BRC | Homozygous *PMS2* c.1500delC | FS | **[5]** | Coding sequence variation resulting in a stop codon i.e. a nonsense or frameshift alteration that is not after codon 743 in *MLH1* or after codon 888 in *MSH2*, and not in the last exon of *MSH6* or *PMS2*. |  |
| 96 | *MSH6* c.1084C>T p.(Pro362Ser) | (MS)/SS | [3] | Low prior probability of pathogenicity as a MS, but some evidence in silico that variant may instead affect splicing. Isolated IHC loss of *MSH6* is good evidence of pathogenicity (apparent lack of MSI-H in *MSH6* tumours is a known test insensitivity). Older age and lack of significant FH is evidence against. | Dual heterozygosity might represent a case of MINAS, as per Whitworth, J, et al. "Multilocus inherited neoplasia alleles syndrome: a case series and review." JAMA oncology 2.3 (2016): 373-379. Each variant on its own may not be wholly pathogenic, but together the two variants act as one that is pathogenic. (Dr Ian Frayling, pers. commun.) |
|  | AND *MSH6* c.2018C>T p.(Pro673Leu) | MS | Not Known | Low prior as a MS. However consistent IHC |  |
| 72 | *MSH6* c.1379G>A p.(Gly460Asp) | MS | [3] | High prior probability of pathogenicity as a MS, so not much evidence would be needed to achieve >0.95, but lack of abnormal IHC/MSI-H, and significant FH. |  |
| 121 | *MSH2* c.569T>C p.(Leu190Pro) | MS | [3] | High prior probability of pathogenicity as a MS, so not much evidence would be needed to achieve >0.95. MMRd and MSI-H in one tumour, but unconvincing FH and no data from other tumours/family members as to segregation. (High BMI in this context argues against LS.) |  |
| 152 | *MSH6* c.1153_delAGG p. (Arg385del) het | ID | 3 | Reported in Roncari et al. (200); PMID 17718861. Class 3 because currently "insufficient evidence", so mosaic MSH6 loss on IHC may be further evidence for pathogenicity, as may segregation data given FH consistent with LS. (Apparent lack of MSI-H in *MSH6* tumours is a known test insensitivity). | Evidence from study of de novo LS cases suggests small indel mutations in MMR genes may be more pathogenic/penetrant than hitherto considered. Overall, 0.54% (8/1486) of all Class 4/5 variants causative of LS are ID, and 2.7% (230/8388) of LS cases reported to the LOVD have ID, whereas 2.4-fold more (4/62: 6.5%) of de novo LS cases have IDs (p = 0.078: Fisher's Exact). (Dr Ian Frayling, personal communication.) |

Abbreviations MS: Missense, SS: Splice site, NS: Nonsense, FS: Frame shift; ID: inDel
