# Supplementary material for: The proportion of endometrial tumours associated with Lynch syndrome (PETALS): A prospective cross-sectional study
Source: PLoS Med. 2020 Sep 17;17(9):e1003263. doi: 10.1371/journal.pmed.1003263 (PMC7497985; doi:10.1371/journal.pmed.1003263)
Supplement: S3 Text — MMR, mismatch repair. (DOCX) [file pmed.1003263.s006.docx]

**The Proportion of Endometrial Tumours Associated with Lynch Syndrome:**

**a prospective diagnostic test accuracy study of unselected screening of endometrial cancer for Lynch syndrome (PETALS study)**

**Supporting Information**

Table of Contents

Appendix 3 2

Germline results 2

Table 6 Demographic details of *VUS_MMR* carriers. 2

Table 7 Familial and molecular details of *VUS_MMR* carriers. 3

# **Appendix 3**

## **Germline results**

### Table 6 Demographic details of *VUS_MMR* carriers.

| **PET ID** | **Grade** | **FIGO Stage** | **Histotype** | **Age** | **BMI** | **Ethnicity** |
| --- | --- | --- | --- | --- | --- | --- |
| 84 | 1 | II | EEC | 25-29 | 36 | Asian |
| 73 | 2 | II | EEC | 30-34 | 58 | White |
| 152 | NA | NA | AEH | 40-44 | 20 | White |
| 101 | 1 | II | EEC | 60-64 | 38 | Asian |
| 72 | 1 | 1a | EEC | 50-54 | 21 | White |
| 121 | 2 | 1a | EEC | 70-74 | 40 | White |
| 116 | 2 | 1a | EEC | 75-79 | 32 | White |
| 157 | 2 | 1a | EEC | 65-69 | 47 | White |
| PREC08 | 1 | 1b | EEC | 70-74 | 31 | White |
| 209 | 2 | II | EEC | 40-44 | 20 | White |
| 150 | 1 | II | EEC | 70-74 | 36 | White |

Abbreviations: FIGO: The International Federation of Gynecology and Obstetrics, EEC: Endometrial Endometrioid Cancer, AEH: Atypical Endometrial Hyperplasia

### Table 7 Familial and molecular details of *VUS_MMR* carriers.

| **PET ID** | **Amsterdam-II Criteria** | **Revised Bethesda Criteria** | **PREMM_5_ Model (>2.5% significant)** | **IHC** | **MSI** | **Mutation** | **InSiGHT Class** | **ACMG class** |
| --- | --- | --- | --- | --- | --- | --- | --- | --- |
| 72 | No | No | 3.3 | Normal | MSS | *MSH6* c.1379G>A p.(Gly460Asp) | 3* | Not Known ( in silico suggest LP) |
| 73 | No | No | 3.3 | Normal | MSS | *MSH2* c.1760-7delT | 3* | VUS |
| 84 | No | No | 4.7 | Normal | MSS | *MSH2* c.499G>C p.(Asp167His) | 1 | VUS |
| 101 | No | No | 2.6 | MLH1/PMS2 Loss (Normal methylation) | MSI-H | *MSH6* c.2018C>T p.(Pro673Leu) | 3* | Not Known ( in silico suggest LP) |
| 116 | No | No | 1.8 | MLH1/PMS2 Loss (Hypermethylation) and MSH6 loss | MSI-H | *MSH6* c.2633T>C p.(Val878Ala) | 1 | LB |
| 121 | No | No | 2.7 | MSH2/MSH6 loss | MSI-H | *MSH2* c.569T>C p.(Leu190Pro) | 3* | Not Known ( in silico suggest LP) |
| 150 | No | No | 2.9 | MSH6 isolated loss | MSS | *MSH6* c.2375T>C p.(Leu792Pro) | 3 | VUS |
| 152 | No | No | 7.1 | Mosaic MSH6 loss | MSS | *MSH6* c.1153_1155del p.Arg385del | 3* | VUS |
| 157 | No | No | 3 | MSH6 isolated loss | MSS | *MSH6* c.1526T>Cp.(Val509Ala) | 2 | LB |
| 209 | Yes | Yes | 1.7 | Normal | MSS | *MSH6* c.3600 A>G p.(lle1200Met) | 3* | VUS |
| PREC08 | No | No | 2.2 | MLH1/PMS2 loss | MSS | *MSH6* c.-118G>A | 3 | VUS |

Abbreviations: IHC: Immunohistochemistry, MSI-H Microsatellite instability, InSiGHT:  International Society for Gastrointestinal Hereditary Tumours, ACMG: American College of Medical Genetics and Genomics, MSS: Microsatellite stable, MSI-H: Microsatellite-High. * Classification provided by expert opinion of Dr Ian Frayling of InSiGHT in private correspondence, LP: Likely pathogenic, VUS: variant of uncertain significance, LB, Likely benign.
