# Supplementary material for: The proportion of endometrial tumours associated with Lynch syndrome (PETALS): A prospective cross-sectional study
Source: PLoS Med. 2020 Sep 17;17(9):e1003263. doi: 10.1371/journal.pmed.1003263 (PMC7497985; doi:10.1371/journal.pmed.1003263)
Supplement: S4 Text — MMR, mismatch repair. (DOCX) [file pmed.1003263.s007.docx]

**The Proportion of Endometrial Tumours Associated with Lynch Syndrome:**

**a prospective diagnostic test accuracy study of unselected screening of endometrial cancer for Lynch syndrome (PETALS study)**

**Supporting Information**

Table of Contents

Appendix 4 2

Somatic results 2

Table 8 Demographic details of somatic *path_MMR* carriers. 2

Table 9 Family history details of somatic *path_MMR* carriers 3

Table 10 Molecular details of somatic *path_MMR* carriers 4

# **Appendix 4**

## **Somatic results**

### Table 8 Demographic details of somatic *path_MMR* carriers.

| **PET ID** | **Age** | **BMI** | **Grade** | **FIGO Stage** | **Histotype** | **Ethnicity** |
| --- | --- | --- | --- | --- | --- | --- |
| 62 | 60-64 | 34 | 3 | IIIC | EEC | White |
| 68 | 65-69 | 24 | 3 | IB | EEC | White |
| 91 | 50-54 | 27 | 3 | IIIB | EEC | White |
| 115 | 65-69 | 24 | 2 | IA | EEC | Asian |
| 139 | 60-64 | 26 | 3 | IA | Undifferentiated | White |
| 150 | 70-74 | 36 | 1 | II | EEC | White |
| 157 | 65-69 | 47 | 2 | IA | EEC | White |
| 182 | 75-79 | 25 | 3 | IA | CarC | White |
| 186 | 60-64 | 21 | 1 | IB | EEC | White |
| 197 | 85-89 | 25 | 2 | IA | EEC | White |
| 258 | 55-59 | 22 | 1 | IA | EEC | Asian |
| 326BRC | 60-64 | 38 | 1 | IA | EEC | White |
| 416BRC | 55-59 | 28 | 2 | IB | EEC | White |
| 44BRC | 55-59 | 28 | 3 | IA | EEC | White |
| 816BRC | 60-64 | 23 | 3 | IB | EEC | White |
| MET014 | 50-54 | 31 | 3 | IIIB | Mixed (EEC and Serous) | Asian |

Abbreviations: FIGO: The International Federation of Gynecology and Obstetrics, EEC: Endometrial Endometrioid Cancer, CarC: Carcinosarcoma

### Table 9 Family history details of somatic *path_MMR* carriers

| **PET ID** | **Amsterdam-II Criteria** | **Revised Bethesda Criteria** | **PREMM_5_ Model (>2.5% significant)** |
| --- | --- | --- | --- |
| 62 | No | No | 2.60% |
| 68 | No | Yes | 8.00% |
| 91 | No | No | 3.00% |
| 115 | No | No | 2.40% |
| 139 | No | No | 2.40% |
| 150 | No | No | 2.90% |
| 157 | No | No | 3.00% |
| 182 | No | No | 2.10% |
| 186 | No | No | 2.50% |
| 197 | No | No | 1.50% |
| 258 | No | No | 2.70% |
| 326BRC | No | No | 2.80% |
| 416BRC | No | No | 3.80% |
| 44BRC | No | No | 4% |
| 816BRC | No | No | 3.00% |
| MET014 | No | No | 3.80% |

### Table 10 Molecular details of somatic *path_MMR* carriers

| **PET ID** | **IHC** | **MSI** | **Germline analysis** | **Somatic analysis (variant frequency)** | **ACMG class ^** | **Additional information (somatic analysis)** |
| --- | --- | --- | --- | --- | --- | --- |
| 62 | MSH6 isolated loss | MSI-H | No mutation | *MSH6* c.3261delC p.(Phe1088SerfsTer2) (10.13%) | P | Mean coverage 2256x (percentage of the coding region covered at 350X read depth; *MLH1* 100%, *MSH2* 96%, *MSH6* 94.5%).  LOH: *MSH2* no evidence, **LOH: *MSH6* Yes**, LOH: *MLH1* uninformative. **Pathogenic** *PTEN* c.697C>T p.(Arg233Ter) 8.75% *PTEN* c.389G>A p.(Arg130Gln) 8.54% *MUTYH* c.1014G>C p.(Gln338His) HOM *CTNNB1* c.313G>A p.(Glu105Lys) 11.61% *APC* c.8195A>G p.(Asp2732Gly) 9.23% *POLE* c.4901G>A p.(Arg1634His) 9.43% *POLE* c.4027G>A p.(Gly1343Ser) 7.86% *POLD1* c.1620C>T p.(Gly540Gly) 60.03% |
| 68 | MSH2/MSH6 loss | MSI-H | No mutation | *MSH2* c.1030C>T p.(Gln344Ter) (15.73%) & *MSH2* c.2377C>T p.(Gln793Ter) (22.72%) | P | Mean coverage 1664x (percentage of the coding region covered at 350X read depth; *MLH1* 97.6%, *MSH2* 96.4%, *MSH6* 89.2%). LOH: *MSH2* no evidence, LOH: *MSH6* no evidence, LOH: *MLH1* no evidence  **VUS** *MUTYH* c.1377C>A p.(Ala459Ala) 4.36% *MLH1* c.1608T>C p.(Pro536Pro) 4.73% *CTNNB1* c.-48-3T>A 4.59% *CTNNB1* c.1674G>A p.(Gln558Gln) 4.22% *APC* c.2459C>A p.(Thr820Asn) 4.92% *APC* c.2850T>G p.(Pro950Pro) 4.62% *APC* c.6209G>A p.(Gly2070Asp) 4.79% *APC* c.7163C>T p.(Ala2388Val) 5.67% *PTEN* c.743C>A p.(Pro248His) 4.94% *POLE* c.5559C>T p.(Ile1853Ile) 4.01% *POLE* c.3614C>T p.(Pro1205Leu) 4.98% *POLE* c.843G>A p.(Leu281Leu) 5.3% *POLE* c.72C>T p.(Gly24Gly) 5.31% *SMAD4* c.573G>A p.(Ser191Ser) 5.44% *POLD1* c.2430G>A p.(Ala810Ala) 5.18% |
| 91 | Patchy loss of MSH2 | MSS | No mutation | *MSH2* c.832G>T p.(Glu278Ter) (30.44%) & *MSH2* c.*3A>C (23.54%) | P | Mean coverage 3048x (percentage of the coding region covered at 350X read depth; *MLH1* 100%, *MSH2* 96%, *MSH6* 93.5%).  LOH: *MSH2* no evidence, LOH: *MSH6* no evidence, LOH: *MLH1* no evidence. **Pathogenic**  *PTEN* c.634+2T>G 35.75% *APC* c.875delT p.(Leu292Ter) 15.52% *APC* c.4729G>T p.(Glu1577Ter) 5.32% *MSH6* c.1082G>A p.(Arg361His) 9.7% |
| 115 | Patchy loss of MSH2/MSH6 | MSS | No mutation | *MSH2* c.1837A>C p.(Asn613His) (51.92%) | VUS or LP | Mean coverage 3495x (percentage of the coding region covered at 350X read depth; *MLH1* 77.3%, *MSH2* 86.1%, *MSH6* 87.8%).  LOH: *MSH2* no evidence, LOH: *MSH6* no evidence, LOH: *MLH1* no evidence. **Pathogenic** *PTEN* c.740T>A p.(Leu247Ter) 27.72% *MUTYH* c.1014G>C p.(Gln338His) HET *POLE* c.6056C>A p.(Ala2019Asp) 48.72% |
| 139 | Patchy loss of MSH2/MSH6 | MSS | No mutation | *MSH6* c.1630G>T p.(Glu544Ter) (5.04%)4 | LP | Mean coverage 4001x (percentage of the coding region covered at 350X read depth; *MLH1* 100%, *MSH2* 96%, *MSH6* 92.6%). LOH: *MSH2* no evidence, LOH: *MSH6* no evidence, LOH: *MLH1* no evidence **VUS** *MUTYH* c.1377C>A p.(Ala459Ala) 4.36% *MLH1* c.1608T>C p.(Pro536Pro) 4.73% *CTNNB1* c.-48-3T>A 4.59% *CTNNB1* c.1674G>A p.(Gln558Gln) 4.22% *APC* c.2459C>A p.(Thr820Asn) 4.92% *APC* c.2850T>G p.(Pro950Pro) 4.62% *APC* c.6209G>A p.(Gly2070Asp) 4.79% *APC* c.7163C>T p.(Ala2388Val) 5.67% PTEN c.743C>A p.(Pro248His) 4.94% POLE c.5559C>T p.(Ile1853Ile) 4.01% POLE c.3614C>T p.(Pro1205Leu) 4.98% POLE c.843G>A p.(Leu281Leu) 5.3% POLE c.72C>T p.(Gly24Gly) 5.31% SMAD4 c.573G>A p.(Ser191Ser) 5.44% POLD1 c.2430G>A p.(Ala810Ala) 5.18%" |
| 150 | MSH6 isolated loss | MSS | VUS: MSH6 c.2375T>C p.(Leu792Pro) | *MSH6* c.2375T>C p.(Leu792Pro) (50.58%) & *MSH6* c.3261delC p.(Phe1088SerfsTer2) (36.74%) | P | Mean coverage 1479x (percentage of the coding region covered at 350X read depth; *MLH1* 100%, *MSH2* 95.7%, *MSH6* 89.0%). LOH: *MSH2* no evidence, LOH: *MSH6* no evidence, LOH: *MLH1* no evidence *MUTYH* c.1014G>C p.(Gln338His) HET **Pathogenic** *PTEN* c.697C>T p.(Arg233Ter) 78.51% *PTEN* c.517C>T p.(Arg173Cys) 37.66% **VUS** *POLE* c.834G>A p.(Thr278Thr) 39.28% |
| 157 | MSH6 isolated loss | MSS | VUS: *MSH6* c.1526T>C p.(Val509Ala) Class 2 | *MSH2* c.2458+1G>A (5.04%) | P | Mean coverage 2590x (percentage of the coding region covered at 350X read depth; *MLH1* 100%, *MSH2* 96.4%, *MSH6* 92.6%). LOH: *MSH2* no evidence, LOH: *MSH6* no evidence, LOH: *MLH1* no evidence. **Pathogenic** *MUTYH* c.1014G>C p.(Gln338His) HET *PTEN* c.634+1G>T 18.5% *PTEN* c.388C>G p.(Arg130Gly) 24.57% **VUS** *MSH6* c.1526T>C p.(Val509Ala) 48.08% *SMAD4* c.1146C>T p.(His382His) 4.48% *POLD1* c.1242+5G>A 5.59% *POLD1* c.1275C>T p.(Ala425Ala) 43.9% *POLD1* c.2073G>A p.(Leu691Leu) 8.64% |
| 182 | MSH6 isolated loss | MSS | No mutation | *MSH6* c.3243_3253del p.(Leu1081PhefsTer8) (44.39%) & *MSH6* c.3589delA p.(Thr1197LeufsTer19) (4.66%) | P | Mean coverage 2325x (percentage of the coding region covered at 350X read depth; *MLH1* 97.6%, *MSH2* 96%, *MSH6* 90.5%). LOH: *MSH2* no evidence, LOH: *MSH6* no evidence, LOH: *MLH1* uninformative **Pathogenic** *APC* c.847C>T p.(Arg283Ter) 50.52% *APC* c.4666dupA p.(Thr1556AsnfsTer3) 47.21% **VUS** *POLD1* c.1294C>T p.(Arg432Trp) 47.58%" |
| 186 | MLH1/PMS2 loss | MSI-H | No mutation | *MLH1* c.968_1000delinsATG p.(Leu323_Leu334delinsHisVal) (10.5%) & *MLH1* c.1852_1854delAAG p.(Lys618del) (22.66%) | P | Mean coverage 3764x (percentage of the coding region covered at 350X read depth; *MLH1* 100%, *MSH2* 97%, *MSH6* 94.8%). LOH: *MSH2* no evidence, LOH: *MSH6* no evidence, LOH: *MLH1* uninformative. **Pathogenic** *PTEN* c.800delA p.(Lys267ArgfsTer9) 14.83% *PTEN* c.955_958delACTT p.(Thr319Ter) 12.79% |
| 197 | MSH2/MSH6 loss | MSI-H | No mutation | *MSH2* c.1147C>T p.(Arg383Ter) 30.39% & *MLH1* c.1852_1854delAAG p.(Lys618del) (16.27%) & *MSH6* c.3261dupC p.(Phe1088LeufsTer5) (9.77%) | P | Mean coverage 3573x (percentage of the coding region covered at 350X read depth; *MLH1* 100%, *MSH2* 97.3%, *MSH6* 92.8%). LOH: *MSH2* yes, LOH: *MSH6* yes, LOH: *MLH1* no evidence. **Pathogenic** *POLE* c.6453C>T p.(Tyr2151Tyr) 51.36% |
| 258 | Patchy loss MLH1/PMS2 | MSS | No mutation | *MLH1* c.2149G>T p.(Glu717Ter) (8.01%) & *MLH1* c.1990-3C>G (9.1%) | P | Mean coverage 2067x (percentage of the coding region covered at 350X read depth; *MLH1* 100%, *MSH2* 96%, *MSH6* 94.3%). LOH: *MSH2* no evidence, LOH: *MSH6* no evidence, LOH: MLH1 no evidence **Pathogenic** *PTEN* c.68T>A p.(Leu23Ter) 8.89% *MSH6* c.3261dupC p.(Phe1088LeufsTer5) 7.85% *PTEN* c.800delA p.(Lys267ArgfsTer9) 7.65% **VUS** *MSH6* c.1178C>T p.(Ala393Val) 6.87% *POLD1* c.1061C>T p.(Ala354Val) 8.76% |
| 326BRC | MSH2/MSH6 loss | MSI-H | No mutation | *MSH6* c.3477delC p.(Tyr1159Ter) (9.84%) & *MSH6* c.3261delC p.(Phe1088SerfsTer2) (17.7%) | P | Mean coverage 2771x (percentage of the coding region covered at 350X read depth; *MLH1* 100%, *MSH2* 96.4%, *MSH6* 92.5%). LOH: *MSH2* no evidence, LOH: *MSH6* no evidence, LOH: MLH1 no evidence **Pathogenic** *POLD1* c.347delC p.(Pro116HisfsTer53) 16.16% **VUS** *MSH6* c.2419G>A p.(Glu807Lys) 12.79% *MLH1* c.-86G>A 11.63% *APC* c.3354T>A p.(Asn1118Lys) 12.9% *APC* c.5122G>A p.(Val1708Ile) 10.95% *POLD1* c.673C>T p.(Arg225Cys) 26.47% |
| 416BRC | Normal | MSI-H | No mutation | *MSH2* c.1864_1865delinsT p.(Pro622Ter) 32.46% & *MSH2* c.1145_1160del p.(Arg382LeufsTer25) (35.88%) & *MSH6* c.3202C>T p.(Arg1068Ter) (9.53%) & *MSH6* c.3261delC p.(Phe1088SerfsTer2) (4.22%) | P | Mean coverage 2605x (percentage of the coding region covered at 350X read depth; *MLH1* 100%, *MSH2* 96%, *MSH6* 93.7%). LOH: *MSH2* no evidence, LOH: *MSH6* no evidence, LOH: *MLH1* no evidence **VUS** *MSH6* c.2941A>T p.(Ile981Phe) 11.11% *MLH1* c.212A>G p.(Glu71Gly) 4.99% *CTNNB1* c.1176A>G p.(Ala392Ala) 7.97% *APC* c.688C>T p.(Arg230Cys) 26.56% *APC* c.4768A>G p.(Lys1590Glu) 9.79% *APC* c.5097G>A p.(Glu1699Glu) 5.64% *POLD1* c.2246C>T p.(Ala749Val) 4.43% |
| 44BRC | MSH6 isolated loss | MSI-H | No mutation | *MSH6* c.560A>C p.(Lys187Thr) (11%) & *MSH6* c.1602del p.(Tyr535Thrfs*36) (13%) | P | Mean coverage 2984x (percentage of the coding region covered at 350X read depth; MLH1 100%, MSH2 96%, MSH6 93.5%).  LOH: MSH2 no evidence, LOH: MSH6 no evidence, LOH: MLH1 no evidence. **Pathogenic**  *PTEN* c.741dupA (p.Pro248Thrfs) 37.6% *APC* c.423-?_729+?del 12.8% *MUTYH* c.1550_1551delGCinsAG (p.Cys517Ter) 11.2% |
| 816BRC | MSH2/MSH6 loss | MSI-H | No mutation | *MSH6* c.718C>T p.(Arg240*) (13.2%) & *MSH2* c.1077-?_*279+?del (9.7%) | P | Mean coverage 3234x (percentage of the coding region covered at 350X read depth; MLH1 100%, MSH2 97%, MSH6 94.8%). LOH: MSH2 no evidence, LOH: MSH6 no evidence, LOH: MLH1 No. **Pathogenic** *PTEN* c.968dupA (p.Asn323Lysfs) 72.3% *CTNNB1* c.1876G>T (p.Glu626Ter) 62.3% |
| MET014 | MLH1/PMS2 loss | MSI-H | No mutation | *MLH1* c.546-2A>T (52.4%) | P | Mean coverage 3374x (percentage of the coding region covered at 350X read depth; MLH1 100%, MSH2 97.3%, MSH6 92.5%). LOH: MSH2 no evidence, LOH: MSH6 no evidence, **LOH: MLH1 yes** **Pathogenic** PTEN c.800delA p.(Lys267ArgfsTer9) 52.77% **VUS** POLE c.3645C>T p.(Phe1215Phe) 15.25% POLE c.1739A>G p.(His580Arg) 27.16%" |

Abbreviations: IHC: Immunohistochemistry, MSI-H Microsatellite instability, ACMG: American College of Medical Genetics and Genomics, MSS: Microsatellite stable, MSI-H Microsatellite instability-High. VUS: Variant of unknown significance, P: Pathogenic, LP: Likely pathogenic
