# Supplementary material for: The proportion of endometrial tumours associated with Lynch syndrome (PETALS): A prospective cross-sectional study
Source: PLoS Med. 2020 Sep 17;17(9):e1003263. doi: 10.1371/journal.pmed.1003263 (PMC7497985; doi:10.1371/journal.pmed.1003263)
Supplement: S5 Text — (DOCX) [file pmed.1003263.s008.docx]

**The Proportion of Endometrial Tumours Associated with Lynch Syndrome:**

**a prospective diagnostic test accuracy study of unselected screening of endometrial cancer for Lynch syndrome (PETALS study)**

**Supporting Information**

Table of Contents

Appendix 5 2

Sensitivity and Specificity analysis 2

Table 11: Diagnostic 2x2 tables for IHC and MSI with or without MLH1 methylation testing 2

Table 12: Diagnostic 2x2 tables for Age and BMI 3

Table 13: Diagnostic 2x2 tables for PREMM5, Amsterdam II criteria, Revised Bethesda criteria, and pathological findings 4

Figure 2 Receiver operator curve (ROC) for the various methods of tumour triage for the detection of *path_MMR* in endometrial cancer 5

# **Appendix 5**

## **Sensitivity and Specificity analysis**

### Table 11: Diagnostic 2x2 tables for IHC and MSI with or without MLH1 methylation testing

| **IHC** | **path_MMR** | **Sporadic / VUS** | **Total** |  |
| --- | --- | --- | --- | --- |
| **Test positive** | 16 | 94 | 110 | PPV 0.145 |
| **Test negative** | 0 | 390 | 390 | NPV 1.000 |
| **Total** | 16 | 484 | 500 |  |
|  | Sensitivity 1.000 | Specificity 0.806 |  |  |
|  |  |  |  |  |
| **IHC w/meth** | **path_MMR** | **Sporadic / VUS** | **Total** |  |
| **Test positive** | 16 | 16 | 32 | PPV 0.500 |
| **Test negative** | 0 | 468 | 468 | NPV 1.000 |
| **Total** | 16 | 484 | 500 |  |
|  | Sensitivity 1.000 | Specificity 0.967 |  |  |
|  |  |  |  |  |
| **MSI** | **path_MMR** | **Sporadic / VUS** | **Total** |  |
| **Test positive** | 9 | 80 | 89 | PPV 0.101 |
| **Test negative** | 7 | 404 | 411 | NPV 0.983 |
| **Total** | 16 | 484 | 500 |  |
|  | Sensitivity 0.563 | Specificity 0.835 |  |  |
|  |  |  |  |  |
| **MSI w/meth** | **path_MMR** | **Sporadic / VUS** | **Total** |  |
| **Test positive** | 9 | 16 | 25 | PPV 0.360 |
| **Test negative** | 7 | 468 | 475 | NPV 0.985 |
| **Total** | 16 | 484 | 500 |  |
|  | Sensitivity 0.563 | Specificity 0.967 |  |  |
|  |  |  |  |  |
| **IHC & MSI** | **path_MMR** | **Sporadic / VUS** | **Total** |  |
| **Test positive** | 16 | 101 | 117 | PPV 0.137 |
| **Test negative** | 0 | 383 | 383 | NPV 1.000 |
| **Total** | 16 | 484 | 500 |  |
|  | Sensitivity 1.000 | Specificity 0.791 |  |  |
| **IHC & MSI w/meth** |  |  |  |  |
|  | **path_MMR** | **Sporadic / VUS** | **Total** |  |
| **Test positive** | 16 | 22 | 38 | PPV 0.421 |
| **Test negative** | 0 | 462 | 462 | NPV 1.000 |
| **Total** | 16 | 484 | 500 |  |
|  | Sensitivity 1.000 | Specificity 0.955 |  |  |

### Table 12: Diagnostic 2x2 tables for Age and BMI

| **Age** | **path_MMR** | **Sporadic / VUS** | **Total** |  |
| --- | --- | --- | --- | --- |
| **< 50 years** | 7 | 61 | 68 | PPV 0.103 |
| **≥ 50 years** | 9 | 423 | 432 | NPV 0.979 |
| **Total** | 16 | 484 | 500 |  |
|  | Sensitivity 0.438 | Specificity 0.874 |  |  |
|  |  |  |  |  |
| **Age** | **path_MMR** | **Sporadic / VUS** | **Total** |  |
| **< 60 years** | 9 | 166 | 175 | PPV 0.051 |
| **≥ 60 years** | 7 | 318 | 325 | NPV 0.978 |
| **Total** | 16 | 484 | 500 |  |
|  | Sensitivity 0.563 | Specificity 0.657 |  |  |
|  |  |  |  |  |
| **Age** | **path_MMR** | **Sporadic / VUS** | **Total** |  |
| **< 70 years** | 15 | 314 | 329 | PPV 0.046 |
| **≥ 70 years** | 1 | 170 | 171 | NPV 0.994 |
| **Total** | 16 | 484 | 500 |  |
|  | Sensitivity 0.938 | Specificity 0.351 |  |  |
|  |  |  |  |  |
| **BMI** | **path_MMR** | **Sporadic / VUS** | **Total** |  |
| **< 35 kg/m²** | 13 | 301 | 314 | PPV 0.041 |
| **≥ 35 kg/m²** | 2 | 182 | 184 | NPV 0.989 |
| **Total** | 15 | 483 | 498 |  |
|  | Sensitivity 0.867 | Specificity 0.377 |  |  |
|  |  |  |  |  |
| **BMI < 35 & Age:** | **path_MMR** | **Sporadic / VUS** | **Total** |  |
| **< 50 years** | 5 | 25 | 30 | PPV 0.167 |
| **≥ 50 years** | 10 | 458 | 468 | NPV 0.979 |
| **Total** | 15 | 483 | 498 |  |
|  | Sensitivity 0.333 | Specificity 0.948 |  |  |
|  |  |  |  |  |
| **BMI < 35 & Age:** | **path_MMR** | **Sporadic / VUS** | **Total** |  |
| **< 60 years** | 7 | 87 | 94 | PPV 0.074 |
| **≥ 60 years** | 8 | 396 | 404 | NPV 0.980 |
| **Total** | 15 | 483 | 498 |  |
|  | Sensitivity 0.467 | Specificity 0.820 |  |  |
|  |  |  |  |  |
| **BMI < 35 & Age:** | **path_MMR** | **Sporadic / VUS** | **Total** |  |
| **< 70 years** | 12 | 174 | 186 | PPV 0.065 |
| **≥ 70 years** | 3 | 309 | 312 | NPV 0.990 |
| **Total** | 15 | 483 | 498 |  |
|  | Sensitivity 0.800 | Specificity 0.640 |  |  |

###

### Table 13: Diagnostic 2x2 tables for PREMM5, Amsterdam II criteria, Revised Bethesda criteria, and pathological findings

| **PREMM_5_** | **path_MMR** | **Sporadic / VUS** | **Total** |  |
| --- | --- | --- | --- | --- |
| **≥ 2.5%** | 11 | 153 | 164 | PPV 0.067 |
| **< 2.5%** | 2 | 133 | 136 | NPV 0.985 |
| **Total** | 13 | 286 | 299 |  |
|  | Sensitivity 0.846 | Specificity 0.465 |  |  |
|  |  |  |  |  |
| **Amsterdam II** | **path_MMR** | **Sporadic / VUS** | **Total** |  |
| **Met** | 4 | 3 | 7 | PPV 0.571 |
| **Not met** | 9 | 284 | 293 | NPV 0.969 |
| **Total** | 13 | 287 | 300 |  |
|  | Sensitivity 0.308 | Specificity 0.990 |  |  |
|  |  |  |  |  |
| **Revised Bethesda** |  |  |  |  |
|  | **path_MMR** | **Sporadic / VUS** | **Total** |  |
| **Met** | 5 | 4 | 9 | PPV 0.556 |
| **Not met** | 8 | 283 | 291 | NPV 0.973 |
| **Total** | 13 | 287 | 300 |  |
|  | Sensitivity 0.385 | Specificity 0.986 |  |  |
|  |  |  |  |  |
| **Histological type** | **path_MMR** | **Sporadic / VUS** | **Total** |  |
| **Endometrioid** | 11 | 340 | 351 | PPV 0.031 |
| **Other** | 5 | 144 | 149 | NPV 0.966 |
| **Total** | 16 | 484 | 500 |  |
|  | Sensitivity 0.688 | Specificity 0.298 |  |  |
|  |  |  |  |  |
| **Tumor-infiltrating lymphocytes** |  |  |  |  |
|  |  |  |  |  |
|  | **path_MMR** | **Sporadic / VUS** | **Total** |  |
| **High** | 15 | 113 | 128 | PPV 0.117 |
| **Normal** | 1 | 371 | 372 | NPV 0.997 |
| **Total** | 16 | 484 | 500 |  |
|  | Sensitivity 0.938 | Specificity 0.767 |  |  |

###
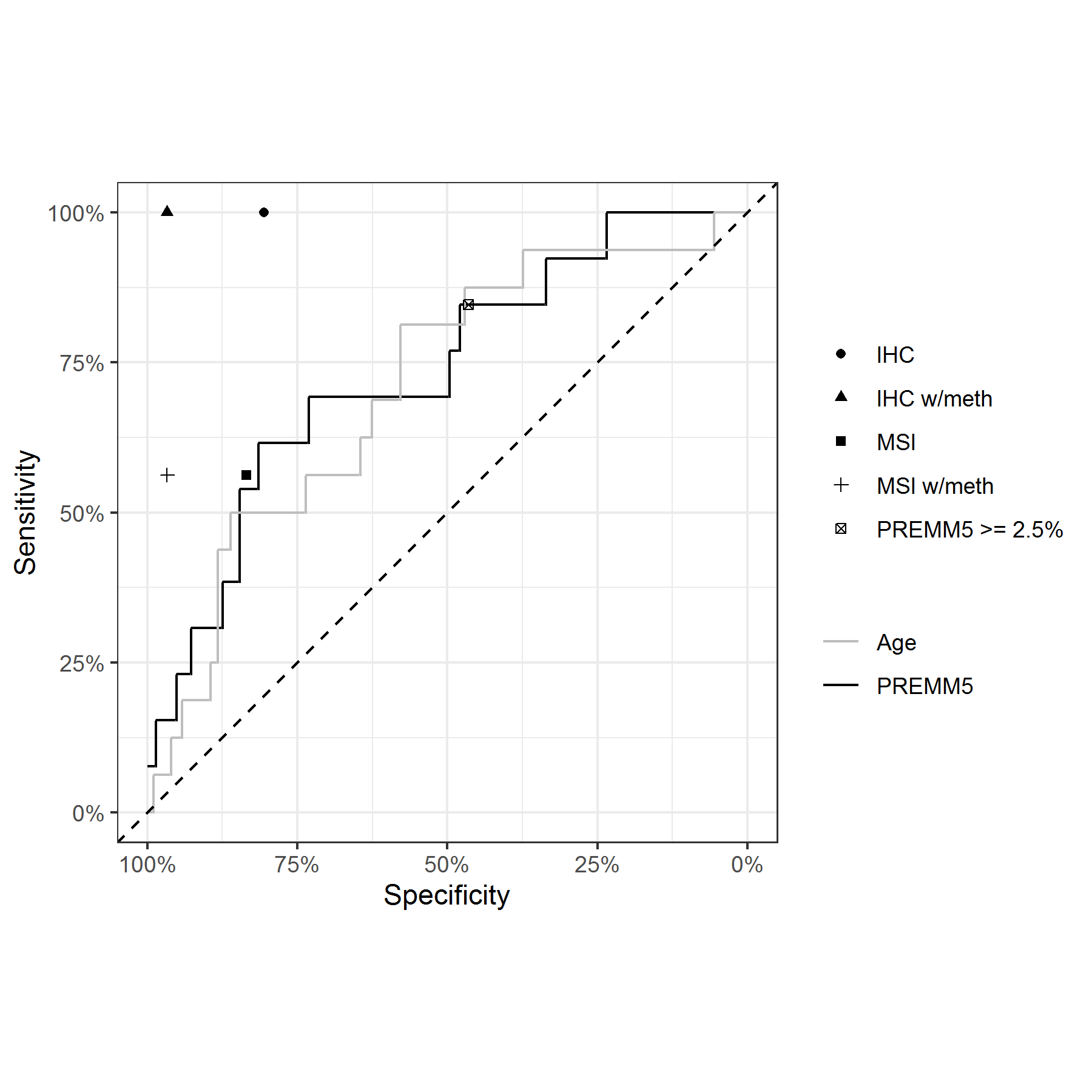


### Figure 2 Receiver operator curve (ROC) for the various methods of tumour triage for the detection of *path_MMR* in endometrial cancer

The trade-off between sensitivity and specificity is demonstrated for age and PREMM_5_ as thresholds are varied (2.5% threshold is highlighted), and symbols show the point estimates for sensitivity and specificity of tumour tests across the study population. The dashed line represents sensitivity + specificity = 1, i.e., a test with no diagnostic utility.
